# Effect of short-term exercise with different programs on prevention of sarcopenia in postmenopausal women: A Quasi-Randomized Controlled Trial

**DOI:** 10.1371/journal.pone.0333171

**Published:** 2025-09-30

**Authors:** Neng Pan, Katarzyna Krasowska, Ossowski Zbigniew

**Affiliations:** Faculty of Physical Culture, Gdansk University of Physical Education and Sport, Gdansk, Poland; Erzurum Technical University: Erzurum Teknik Universitesi, TÜRKIYE

## Abstract

**Background:**

Physical exercise is an effective measure to prevent sarcopenia. However, the effects of Nordic walking based on high-intensity interval training (HIIT NW) and conventional strength training (ST) on the parameters related to sarcopenia in postmenopausal women remain unclear. Therefore, this study aims to evaluate the effects of 12-week HIIT NW and ST on body composition and physical function performance in postmenopausal women.

**Method:**

The participants were 71 women aged between 60 and 79 years old without sarcopenia. Participants were randomly assigned to the HIIT NW group (12-week Nordic walking training, 3 × /week), the ST group (12-week strength training, 3 × /week), and the control group. The body composition was determined by using Otupole InBody 720. Test the hand grip strength with a digital hand force gauge. The strength of the extensor and flexor muscles of the knee joint was measured using Biodex System 4 Pro™. This study also employed common methods for measuring functional performance and conducted two measurements of blood creatinine and creatine kinase.

**Result:**

Compared with the control group, significant improvements were observed in parameters such as Time Up and Go (TUG) and knee joint flexor strength (KFS) in both the HIIT NW group and the ST group. In the ST group alone, significant enhancements were noted in parameters including walking speed (GS) and hand strength on the left side (HS-L). Following the intervention, the HIIT NW group exhibited a marked increase in limb lean mass, which led to a significant rise in the skeletal muscle index (SMI) (p < 0.001). However, the body fat mass (BFM) and body mass index (BMI) decreased significantly in the ST group (p < 0.001 and p = 0.005, respectively). No significant changes were observed in the control group.

**Conclusion:**

Both HIIT NW and ST interventions can effectively prevent sarcopenia in postmenopausal women. The former focuses on improving lower limb strength, while the latter focuses on improving upper limb strength. In the short term, the HIIT NW intervention model is more beneficial for postmenopausal women with normal weight, while the conventional ST intervention model is more conducive to the overweight population.

## 1. Introduction

The global population is facing the challenge of aging [1–3], and the prevalence of various diseases has become a focal point of concern. Sarcopenia is a skeletal muscle disorder that commonly occurs with advancing age and has a number of long-term effects [4]. According to reviews, up to 29% of older people in a community healthcare environment have sarcopenia [5]. However, few studies have reported the global prevalence of sarcopenia, and there is a high level of heterogeneity between studies [6].

Although global research on sarcopenia faces several controversies—such as inconsistent diagnostic criteria and population heterogeneity — its clinical symptoms and harmful effects continue to warrant significant attention. Sarcopenia is a geriatric condition characterized by progressive loss of muscle mass and function and is associated with various adverse health outcomes [7]. Sarcopenia increases the risk of falls, fractures, dependency, use of hospital services, institutionalization, poor quality of life, and mortality [8]. In recent years, an increasing number of studies have demonstrated that sarcopenia is associated with neurological diseases [9]. There is growing evidence that a range of problems such as bone health, type 2 diabetes, cancer, obesity, sedentary behavior and physical inactivity can contribute to sarcopenia in older adults [10–14].

One popular treatment for sarcopenia is exercise. In people with sarcopenia, exercise can enhance skeletal muscle mass, strength, and physical function to varied degrees. It is also an effective way to prevent and treat sarcopenia [15]. The best therapies for enhancing quality of life in older persons with sarcopenia were resistance exercise, either alone or in conjunction with nutrition, and resistance exercise combined with aerobic and balance training, according to evidence of high or moderate certainty [16]. In older people with sarcopenia, knee extensor strength (KES) and gait speed (GS) can be improved by resistance training (RT) and metabolic training (MT), but not by whole body vibration training (WBVT). All three training modes improved time up and go (TUG) times, but did not improve chair stand (CS) times [17]. As the above points demonstrate the effectiveness of exercise for sarcopenia, attention should be focused on the type, dose, and composition of exercise. Strength training is considered an effective treatment for senile muscular osteoporosis [18] and can improve muscle strength and mass. At the same time, simpler, less expensive, or more fun methods are also being studied, one of which is Nordic walking.

Nordic walking (NW) is an easy physical exercise that is usually recommended for clinical populations and the elderly [19]. The advantages of NW training and its effects on sarcopenia need to be clarified. NW training provides additional benefits to the upper body compared to regular walking training, and it allows people to maintain their balance while walking and prevents falls [20,21], which is a good option for older people who have experienced a fear of walking after falling. Regarding the effect of NW on sarcopenia, researchers who compared Nordic walking with multi-component training concluded that moderate-intensity continuous training (MCT) intervention had no advantage over NW training. One possible reason is that NW intervention not only improves cardiovascular capacity but also improves motor capacity, including coordination [22]. It is worth mentioning that NW training has a positive effect on strength improvement and studies have demonstrated beneficial effects on the femur index. Although Zbigniew Ossowski et al mentioned in a study that the effect of Nordic walking training on parameters related to sarcopenia in women with low bone mass is unknown [23], this study provides new evidence for this. A meta-analysis also concluded that it is unclear which exercise is most beneficial for people with sarcopenia [24]. In summary, while the precise impact of NW training on sarcopenia remains uncertain, it exhibits strong universality and entails a low implementation cost. In addition, research has demonstrated that it can lead to a reduction in systolic blood pressure and ferritin levels among postmenopausal women [25,26], Moreover, Nordic walking has been associated with increased muscle strength in the limbs [27]. The reason for this study is to investigate whether it is as effective as regular strength training in preventing sarcopenia.

In recent years, high-intensity interval training (HIIT) has attracted considerable attention. Studies have shown that low-load resistance training (LRT) does not improve muscle mass or strength [28]. In contrast, many studies have suggested that high-intensity interval training (HIIT) may be beneficial for the prevention or treatment of sarcopenia in elderly individuals [29,30]. However, a more conservative assessment of this view was made by a systematic review, which found that many preliminary studies had a high risk of bias, and a limited number of studies were flawed [31]. Therefore, this study attempted to combine NW training with the HIIT training guidelines for older adults recommended by the American College of Sports Medicine (ACSM). Notably, this is a study of the effectiveness of a novel mode of exercise (HIIT NW), and the first to compare it with traditional strength training and no exercise.

In the medical industry, the adage “prevention is better than cure” is used. This study hypothesized that both HIIT NW training and ST training would have good effects on preventing sarcopenia in postmenopausal women, and that the two effects might be reflected in different parameters.Therefore, the aim of this quasi-randomized controlled trial is to evaluate the short-term effects of high-intensity interval Nordic walking (HIIT-NW) compared to traditional resistance training and a no-exercise control on sarcopenia-related parameters in postmenopausal women. To investigate how two distinct forms of exercise, doses, and activity schedules can help postmenopausal women avoid developing sarcopenia. Body composition, physical function, and blood markers of muscle strength were among the particular characteristics used to compare the real effects of various activities and their relative benefits and drawbacks. It is important to note that in order to determine the effect of one parameter, this study will perform correlation tests on the various parameter categories mentioned above in an attempt to determine whether the changes among them are related. This will allow us to pay attention to the extent of change of other parameters. This will be useful for verifying the control variables and test parameters in subsequent research of a similar nature.

## 2. Methods

### 2.1. Ethics statement

The research protocol was approved by the Bioethics Committee of the Gdansk Regional Medical Chamber (procedure number KB-5/22). Before the study began, all subjects received an oral and written description of the experiment and signed an informed consent form prior to participation. This study will submit the “Human Participants Research Checklist,” which includes critical information such as the review and approval documents from the ethics committee, as well as the recruitment dates of the participants. Additionally, this study provides a formal statement confirming that informed consent forms signed by all participants have been obtained. The entire study protocol was registered on ClinicalTrials.gov (NCT06718023) under the title ‘Diverse Training Programs and Sarcopenia in Postmenopausal Women’ on December 4th, 2024. Due to the lack of registration experience in this study, clinical registration was not conducted before the start of the experiment. However, clinical registration was completed before data collection and manuscript writing. The authors confirm that all ongoing and related trials for this intervention are registered. This study adheres to the principles of openness, transparency, and reproducibility.

### 2.2. Participants

**Fig 1:**
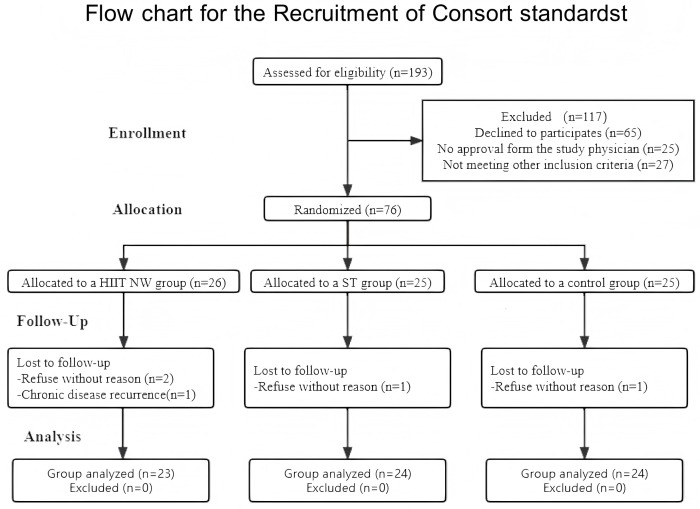
Recruitment process. Note: HIIT NW: NW training based on high-intensity interval training; ST: Strength Training.

The study included 71 postmenopausal women (3 left-handedness) aged 60–79 years (M = 68.89 years, ± 4.07 years), Height (M = 160.02 cm, ± 6.18 cm), Weight (M = 69.11 kg, ± 10.56 kg), BMI (M = 27.13 kg/m^2^, ± 4.14 kg/m^2^) who were Caucasian women. Participants were recruited from Gdansk and Gdynia from October 18, 2022 to January 10, 2023. A total of 76 volunteers aged between 60 and 80 years were selected from an initial pool of 193 women who expressed an interest in participating in the study. The recruitment process is shown in Fig 1. This study combined a pre-test and post-test with a repeated-measures design component to capture changes within the subject over time with quasi-experimental testing to assess differences between groups. Participants were allocated to groups based on their geographical location, controlling for potential environmental and socioeconomic factors that could affect the study’s results. This approach was intended to minimize any differences due to living conditions depending on the city of residence. This quasi-random allocation of participants allowed for more homogeneous groups in terms of important variables, such as the level of daily physical activity and access to health-promoting facilities.

There were 23 cases in the HIIT NW group (M = 69.77 years, ± 4.4 years), 24 patients in the ST group (M = 67.46 years, ± 4.24 years), and 24 patients in the CG group (M = 69.48 years, ± 3.45 years) the above age parameters were based on self-report. Participants in the CG did not participate in any physical activity other than routine activities appropriate to their age, while participants in the HIIT NW and ST groups were asked to participate in a training program three times a week (as specifically mentioned in the study design section).

Participants were included based on the following criteria: postmenopausal women aged over 60 years (i.e., more than 12 months since their last menstrual cycle), absence of contraindications to exercise after the case report and initial diagnosis, and provision of signed informed consent to participate in the study and physical activity program.

Participants were excluded based on the following criteria: self-reported or diagnosed medical conditions that clearly rendered them unsuitable for exercise participation (e.g., uncontrolled hypertension, coronary artery disease, rheumatoid arthritis, type 2 diabetes, respiratory and pulmonary diseases), and unwillingness to adhere to the prescribed schedule.

The participants were required to undergo compulsory medical examinations and provide information regarding prescribed medication usage. Examined women were under the permanent care of a medical specialist who monitored the dosed medicines.

### 2.3. Study design

This study has no deviation from the initial study protocol. It should be noted that the initial study protocol approved by the ethics committee also included several other studies, which were not related to this study. Therefore, this study explores the effects of different exercises in preventing sarcopenia in postmenopausal women based on the initial study protocol. Testing procedures were performed at a single center, and all tests were performed by the same investigators. The NW group intervention was always conducted in the same forest in Gdansk, and the ST group intervention was always conducted in the gym and gymnasium of Gdansk sport university.

#### 2.3.1. HIIT Nordic walking group.

**Fig 2:**
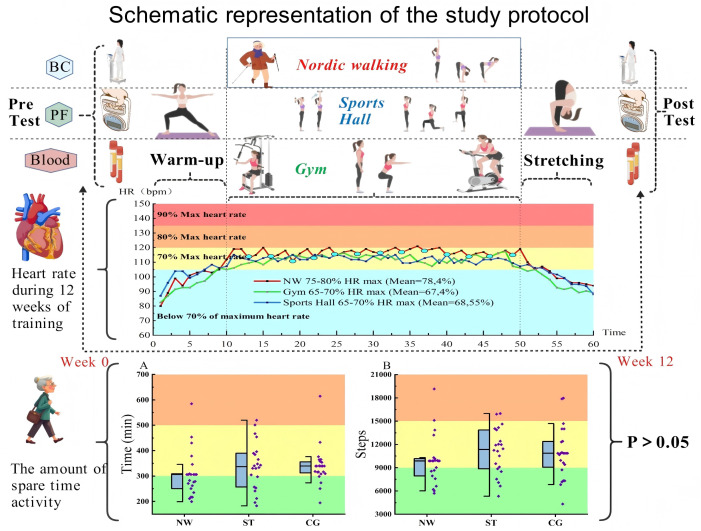
Schematic representation of the study protocol. Note: BC: Body composition; PF: Physical function; HR: Heart rate; P, p-value; A: Walking time per week; B: Steps per week; Blue dots, Nordic walking intervals.

The HIIT NW training sessions were conducted by a certified NW instructor, leading participants in the outdoor forest of Gdansk. Training was 60 minutes each time, 3 times a week, with an interval of 2 days, for a total of 12 weeks. The specific process is illustrated in Fig 2. The participants used a professional Nordic Pole (Exel, Espoo, Finland). According to the recommendations of the International Nordic Walking Association (INWA), the length of the pole depends on the height of the participants (cm) multiplied by 0.68. Because there is a difference of 5 cm between poles of various sizes, this study uses the rounding method [32]. In the first week of training, the main purpose was to improve the participants’ condition during the march, including walking posture, pole usage, breathing rhythm, and lower limb power skills in difficult terrains. As the participants become more skilled, this study only needs to control the exercise dose and adhere to the plan.

Each training session consisted of three phases. During the warm-up period, versatile exercises with poles were performed. The main part of the session was based on 60-second intervals conducted at high intensity at a level of 75–80% (maximum heart rate (HRmax)). The maximum heart rate was calculated as HRmax = 206-0.88 × age [33]. Participants completed 8–13 intervals with a 60-second break. The final part of the session included static and dynamic flexibility exercises with poles. At this point, the participant’s heart rate showed a downward trend and gradually returned to the normal range, which was monitored using a Polar V-800 heart rate monitor (Polar Electro Oy, Finland).

#### 2.3.2. Strength training group.

**Strength Training (ST):** This strength-training program lasted for 12 weeks and was carried out in the gym and sports hall, with three 60-minute sessions per week. The training load strictly follows the resistance training guidelines for the elderly set by the American College of Sports Medicine (ACSM) [34]. Two sessions per week were conducted in the gym with progressive resistance training using dumbbells and professional strength-training equipment. The load intensity was 65–75% of the one-repetition maximum (1-RM), and the exercise intensity was controlled at 60–65% of the maximum heart rate (HR max). As shown in Fig 2, the training was organized in circuit mode, with each session including 10–13 training exercises. Each exercise was performed for one set of 10–15 repetitions, with a 60–90 second rest between exercises. The main training equipment was the HES multistation comprehensive training equipment (model A-25-Sk, Wrocław). In addition, gym training sessions were arranged each week. The training uses 1–4 kg dumbbells, and each movement was completed in two sets (with a 60-second rest between sets) for 10–15 repetitions. The training content systematically covers the upper limbs (arms), lower limbs (legs), trunk, abdomen, and chest muscle groups, including multijoint compound movements and isolated muscle strength training [35,36]. All sessions included standardized warm-up and relaxation segments. The training progression plan was dynamically adjusted based on the individual tolerance and performance of the participants.

#### 2.3.3. Control group.

The control group continued their regular daily activities without participating in targeted physical exercise.

#### 2.3.4. Adherence.

To obtain more realistic results, this study asked participants in all three groups to wear a device that monitors walking data (Polar Watches) and upload each person’s walking time and step count to the corresponding software (Polar FlowSync) once a week. The participants were asked to wear their watches throughout the day, except when necessary (e.g., bathing). The collected data analysis reports were used to review participants in all three groups and did not affect the results by the amount of weekly activity, as shown in Fig 2. It is worth mentioning that no participant missed any session during the intervention period.

### 2.4. Outcome

#### 2.4.1. Primary outcome measures.

Four primary outcome measures were set in this study, including skeletal muscle index (SMI kg/m^2^), skeletal muscle mass (SMM kg), gait speed (GS m/s) and grip strength (HS kg). Two tests were performed, before and after the experiment. A detailed description of each result indicator is provided below.

Sarcopenia is defined by the Asian Sarcopenia Working Group (AWGS) in the 2019 consensus criteria as low muscle mass, low muscle function, or decreased physical performance [37]. The skeletal muscle index (SMI) and skeletal muscle mass (SMM) are often used to evaluate muscle mass. SMI calculates the skeletal muscle content of the limbs divided by the square of the height, and the resulting parameters are compared with established standards [38]. SMI < 8.5 kg/m^2^ in men or <5.75 kg/m^2^ in women may indicate sarcopenia. This study used the InBody 720 (made in Korea) body composition analyzer to measure skeletal muscle content in the extremities as well as skeletal muscle mass (SMM). The accuracy and effectiveness of this device have been recognized by several studies [39]. Measurements were always performed by experts at the Medical University of Gdansk and the data were recorded. Participants were asked to wear as light clothing as possible and to be barefoot during the measurements. Hand grip strength (HS) was used to evaluate muscle function, and a grip strength of less than 28 kg in men or 18 kg in women is considered a risk factor for sarcopenia [40]. Grip strength was measured using a digital hand dynamometer (SAEHAN, Changwon, Korea). To ensure the accuracy of the test, each hand 3 times, with each interval of more than 1 min, taking the maximum of three tests. The main evaluation index for physical performance was gait speed (GS). When the gait speed is less than 1.0 m/s, body function is decreased, which is also a reference marker of sarcopenia [41]. The GS test was carried out indoors, and the best time was needed to cover a distance of 6 m (m/s).

#### 2.4.2. Secondary outcome measures.

The secondary outcome measures set in this study included the dimensions of body composition (BC) and physical function (PF). Some basic body composition indicators can reflect lateral sarcopenia [42]. At the same time, this study sampled several indicators of physical function used to reflect sarcopenia, which are quantitative measures and more objective and persuasive in clinical studies than qualitative measures of self-reporting [43]. The following is a detailed explanation of the test indicators:

The BC consists of Body Fat Mass (BFM kg), Arm Lean Mass (ALM kg), Trunk Lean Mass (TLM kg), Leg Lean Mass (LLM kg) skeletal muscle lean mass (SLM) and fat free body mass (FFM kg). An InBody 720 (made in Korea) body composition analyzer was used to measure these indicators.

PF consists of a Chair Stand (CS times/30s), Arm Curl (AC times/30s), Time Up and Go (TUG), Tandem Balance (TB), knee extensor strength (KES Nm), and knee flexor strength (KFS Nm). CS is widely used as a test index to evaluate physical function in the elderly [44]. This study set the time to 30 s, and the height of the chair used ensured that the knee curvature of the participants was close to 90° ± 5°. The participants were required to complete as many sitting and standing movements as possible within a specified time. Each standing up after sitting was recorded as the complete number of times, and the maximum number of times they completed it was recorded. The AC test is widely used in physical fitness tests for older adults [45]. This study set the time to 30 s and used a 2.27 kg (5 lbs) weight as a resistance object. To reduce compensation in other parts of the body, participants were required to sit in a chair for the entire time, with their backs pressed against the back of the chair and their spine as straight as possible. Participants were encouraged to complete as many bends as possible, recording the maximum number of completions for both their left and right hands. TUG is also an important indicator of physical fitness in the elderly, and can evaluate the lower limb function level of the elderly [46]. First, the participant sat in a chair with a knee curvature close to 90° ± 5°, then stood up and started walking without relying on arm strength after hearing the command, turned 180°, returned to the chair when walking to the 3m mark, and finally sat back in the original chair. The time between when the command was given and when the participant finally sat back in the chair was recorded. TB is a slightly risky test that measures walking balance in older adults [47]. This study use a tape measure on the ground to measure the start and end of the 2 m distance and mark a straight line. Participants were required to walk in this straight line, keeping their heels in line with the tips of their feet as they walked. Record the time from start to finish. KES and KFS are important parameters for evaluating lower limb muscle strength in elderly women [48]. This study used isokinetic peripheral muscle strength tester (Biodex System 4 ProTM) to test the above two indexes. The test is conducted in a heated room with an instructor and a recorder who registers the data. Isometric measurement was performed for the extensors and flexors of the knee joint. Before starting the study, each subject had a 5-minute warm-up on a cycle ergometer. After warm-up, subjects were positioned in the equipment according to the manufacturer’s manual (seated with arms hanging along the body, hands holding the lateral handles, and strap stabilization of trunk, hip, and tested thigh, with the knee flexed to 90°). The isometric strength test was used for three maximal contractions, provided that one single contraction lasted 5 s with 30 s breaks. The seat position was adjusted for the leg length of each tested person. Data were analyzed using the results obtained from the dominant lower limb.

#### 2.4.3. Other outcome measures.

This study also selected some outcome indicators to evaluate sarcopenia, including 2 blood indicators creatinine (Crea mg/dl) and creatine kinase (CK u/l), upper arm circumference (UAC cm), body mass index (BMI kg/m^2^), and muscle strength evaluation index of 2 upper limbs (ULI-1 kg, ULI-2 kg/m^2^) and 2 lower limbs (LLI-1 Nm/kg, LLI-2 Nm/kg). Below is a detailed description of these metrics.

The measurements of creatinine (Crea) and creatine kinase (CK) were performed using a spectrophotometric method on fasting venous blood samples. After collection, the blood was centrifuged to obtain serum. Creatinine concentration was determined by the Jaffé method, where creatinine forms a colored complex with picric acid in an alkaline environment, and the result was measured at a wavelength of 510 nm. CK activity was measured using an enzymatic reaction method, where CK catalyzes the phosphorylation of creatine with ATP, and the resulting ADP was measured at 340 nm. Measurements were performed at 37°C, and the results were expressed in mg/dl for creatinine and U/l for CK. All analyses were conducted on a UV-Vis spectrophotometer, and quality control included measurements of control samples and repeating measurements in the case of hemolysis. Creatinine levels can be affected by reduced muscle mass, and in people with sarcopenia, there is a corresponding decrease in metabolically produced creatinine due to reduced muscle mass, potentially resulting in lower blood creatinine levels [49]. People with sarcopenia have reduced muscle mass and strength, which can lead to muscle damage or inflammation, which can cause increased creatine kinase levels.

UAC can be used as a proxy indicator to evaluate sarcopenia. Muscle strength depends on its physiological cross-section, but it should be noted that changes in arm dimension may also be affected by changes in fat mass. Therefore, attention should be paid to arm lean mass (ALM) when interpreting UAC. [50]. This study employ a customized tape measure to determine upper limb circumference accurately and minimize manual measurement errors by employing a goniometer. There may be a relationship between BMI and sarcopenia, and one study showed that people with sarcopenia tend to be obese [51]. BMI is measured using a formula set in the InBody 720 (made in Korea) instrument (BMI = weight/height^2^). In order to more accurately judge the muscle strength of the limbs, this study used two upper limb indicators: ULI-1(Peak grip strength/BW) and ULI-2 (Peak grip strength/BMI). Two lower limb indexes: LLI-1 (Peak KES/BW), LLI-2 (Peak KFS/BW).

### 2.5. Randomisation

In this study, a list of random numbers was used to assign subjects to HIIT NW, ST, and CG groups in order, and to ensure the same ratio, which was performed by a researcher without knowing the grouping. Because of the nature of the intervention, it is difficult to blind the participants, but the staff involved in the study (the people performing the intervention) and the statistical staff were not aware of the grouping. It is important to highlight that this study did not achieve full randomization due to the phased recruitment of participants. Complete randomization could have introduced potential group differences across various stages. As a result, participants were assigned using a quasi-randomization method.

### 2.6. Statistical methods

Firstly, the following parameters were used in the sample size calculation: statistical test = ANOVA: Repeated measures, within-between interaction; effect size (f) = 0.25; significance level (α) = 0.05; statistical power (1 − β) = 0.80; number of groups = 3; number of measurements = 2; correlation among repeated measures = 0.50. Using G-Power 3.1. Secondly, the preliminary data checking is done in Excel. Then SPSS 27 was used to test the normality, difference, effect size and correlation of the data, and 95% confidence interval was set. The Shapiro-Wilk test was used to check the normal distribution of the data. Data conforming to the normal distribution were subjected to one-way ANOVA and paired sample T-test. Kruskal-Wallis H test was used for data with skewed distribution and multiple comparisons were performed afterwards, and Friedman test was used for intra-group comparison for K relevant samples. The normal distribution data is represented by the mean ± standard deviation, and the skew distribution data is represented by the quartile (with emphasis on the median and outlier). In correlation test, Pearson test was used for data with normal distribution and Spearman test was used for data with skew distribution. In the test of effect size in the same group, Cohen’s d value was used to represent the effect size of data conforming to normal distribution (effect size criteria: small, d = 0.2; Medium, d = 0.5; Large, d = 0.8). The rank biserial correlation coefficient is calculated when dealing with skewed distribution data (effect size criteria: small, r = 0.1; Medium, r = 0.3; Large, r = 0.5). In the test of effect size between groups, the effect size was represented by the value of eta^2^ (effect size criteria: small, ƞ^2^ = 0.01; Medium, ƞ^2^ = 0.06; Large, ƞ^2^ = 0.14). Finally, all images were produced using OriginPro 2024b, RStudio 4.4.2 and GDP online composition software [52].

## 3. Results

### 3.1. Baseline characteristics

A total of 71 people participated in this study, including 23 in HIIT NW group, 24 in ST group and 24 in CG group, and there was no significant difference in distribution. No significant differences were found among groups at baseline for any variables, As shown in Table 1.

**Table 1:** Baseline characteristics.

Outcomes	HIIT NW (n = 23)	ST (n = 24)	CG (n = 24)	p
Height (cm)	159.82 ± 5.88	160 ± 6.1	160.23 ± 6.78	0.974
Age (years)	69.78 ± 4.3	67.46 ± 4.24	69.46 ± 3.37	0.102
Weight (kg)	68.64 ± 9.3	66.84 ± 8.61	71.84 ± 13.01	0.255
**Primary**	**HIIT NW (n = 23)**	**ST (n = 24)**	**CG (n = 24)**	
SMM (kg)	23.15 (20.97 - 24.79)	22.42 (21.18 - 23.56)	23.2 (21.93 - 25.63)	0.481
HS-R (kg)	24.67 ± 2.85	25.07 ± 4.04	26.15 ± 4.37	0.394
HS-L (kg)	24.1 (22.22 - 25.65)	24.55 (21.53 - 27.35)	25.55 (21.9 - 27.88)	0.741
GS (m/s)	1.53 (1.35 - 1.74)	1.57 (1.53 - 1.61)	1.56 (1.42 - 1.74)	0.84
SMI (kg/m^2^)	6.69 (6.23 - 7.14)	6.49 (6.25 - 6.94)	6.59 (6.32 - 7.02)	0.815
**Secondary**	**HIIT NW (n = 23)**	**ST (n = 24)**	**CG (n = 24)**	
BFM (kg)	25.56 ± 6.98	25 ± 7.8	27.71 ± 9.99	0.5
ALM-R (kg)	2.16 ± 0.3	2.24 ± 0.36	2.33 ± 0.35	0.203
ALM-L (kg)	2.13 ± 0.33	2.2 ± 0.34	2.3 ± 0.34	0.255
TLM (kg)	19.43 (18.38 - 20.72)	18.82 (18.16 - 20.83)	20.33 (18.59 - 21.63)	0.468
LLM-R (kg)	6.32 ± 0.92	6.26 ± 0.88	6.3 ± 0.93	0.973
LLM-L (kg)	6.37 ± 0.96	6.31 ± 0.88	6.34 ± 0.94	0.977
SLM (kg)	40.5 (37.95 - 43.05)	39.3 (37.6 - 42.43)	40.5 (38.28 - 44.35)	0.588
FFM (kg)	43.07 (40.45 - 45.75)	41.7 (39.83 - 44.93)	43.11 (40.7 - 47.1)	0.563
CS (times/30s)	16 (13.5 - 18.5)	16.5 (14 – 18 )	16.5 (15 - 17.25)	0.775
TB (s)	4.99 (4.78 - 5.78)	5.18 (4.31 - 5.95)	5.28 (4.73 - 6.67)	0.694
TUG (s)	5.97 (5.74 - 6.45)	6.15 (5.72 - 6.51)	6.31 (5.96 - 7.02)	0.33
AC-R (times/30s)	16 (15 – 18 )	17.5 (16 – 20 )	17 (15.75 - 18.25)	0.53
AC-L (times/30s)	17.19 ± 3.04	18.17 ± 2.14	17.83 ± 3.19	0.491
KES (Nm)	98.18 ± 21.01	102.13 ± 17.87	100.76 ± 26.7	0.826
KFS (Nm)	39.3 (36.25 - 42.25)	42.32 (38.3 - 42.32)	41.47 (37.93 - 43.71)	0.171
**Other**	**HIIT NW (n = 23)**	**ST (n = 24)**	**CG (n = 24)**	
Crea (mg/dl)	0.77 (0.75 - 0.8)	0.76 (0.71 - 0.83)	0.73 (0.69 - 0.77)	0.139
CK (u/l)	73 (59.5 - 92)	85.5 (68 - 103.25)	78.5 (68.5 - 115)	0.552
BMI (kg/m^2^)	26.87 ± 3.36	26.51 ± 4.01	28.01 ± 4.91	0.43
UAC-R (cm)	31.56 ± 2.72	31.32 ± 2.86	32.55 ± 3.86	0.373
UAC-L (cm)	31.46 ± 2.7	31.23 ± 2.87	32.44 ± 3.92	0.391
ULI-1 (kg)	0.37 (0.33 - 0.42)	0.39 (0.35 - 0.44)	0.39 (0.31 - 0.43)	0.701
ULI-2 (kg/m^2^)	0.94 (0.85 - 1.06)	1.04 (0.88 - 1.15)	1.01 (0.77 - 1.15)	0.664
LLI-1 (Nm/kg)	1.49 ± 0.35	1.54 ± 0.33	1.44 ± 0.43	0.636
LLI-2 (Nm/kg)	0.59 ± 0.1	0.63 ± 0.12	0.59 ± 0.15	0.485

Note: SMM: Skeletal muscle mass; HS-R: Hand strength-right; HS-L: Hand strength-left; GS: Gait speed; SMI: Skeletal muscle index; BFM: Body fat mass; ALM-R: Arm lean mass-right; ALM-L: Arm lean mass-left; TLM: Trunk lean mass; LLM-R: Leg lean mass-right; LLM-L: Leg lean mass-left; SLM: Skeletal lean mass; FFM: Fat free mass; CS: Chair stand; TB: Tandem Balance; TUG: “Time up and go” test; AC-R: Armcurl-right; AC-L: Armcurl-left; KES: Knee extensor strength; KFS: Knee flexor strength; Crea: Creatinine; CK: Creatine kinase; BMI: Body mass index; UAC-R: Upper arm circumference-right; UAC-L: Upper arm circumference-left; ULI-1: Upper limbs strength index 1; ULI-2: Upper limbs strength index 2; LLI-1: Lower limb strength index 1; LLI-2: Lower limb strength index 2. Numbers connected by “±” are the mean and standard deviation, numbers before “-” are the median, and numbers connected by “-” are the maximum and minimum values. 95% confidence intervals were used for all data expressed as means and standard deviations.

### 3.2. Comparison between groups after the experiment

The comparison of all outcome parameters in this study is shown in Table 2.

**Table 2:** Comparison of post-experimental outcome measures.

Outcomes	Group			p	Effect size
Primary	HIIT NW (n = 23)	ST (n = 24)	CG (n = 24)		
SMM (kg)	23.70 (28.34 - 20.58)	23.72 (30.74 - 21.77)	23.43 (26.39 - 18.57)	0.245	ƞ^2^ = 0.003
HS-R (kg)	25.30 (35.2 − 23)	27.25 (43.5 - 22.3)	25.13 (32.3 - 19.3)	0.117	ƞ^2^ = 0.011
HS-L (kg)^*^	25.30 (32.8 - 22.4)	25.8 (37.7 - 21.2)^*^	23.92 (29 - 17.4)^*^	0.047	ƞ^2^ = 0.003
GS (m/s)^*^	1.65 (2.08 - 1.41)	1.71 (2.59 - 1.26)^*^	1.49 (1.95 - 1.13)^*^	0.011	ƞ^2^ = 0.124
SMI (kg/m^2^)	6.84 (7.77 - 5.64)	6.57 (8.64 - 5.99)	6.49 (7.86 - 5.71)	0.276	ƞ^2^ = 0.042
**Secondary**	**HIIT NW (n = 23)**	**ST (n = 24)**	**CG (n = 24)**		
BFM (kg)	24.53 ± 7.2	23.4 ± 7.44	28.55 ± 9.61	0.08	ƞ^2^ = 0.02
ALM-R (kg)	2.23 (2.69 - 1.59)	2.23 (3.16 - 2.01)	2.19 (3.1 - 1.67)	0.989	ƞ^2^ = 0.015
ALM-L (kg)	2.20 (2.87 - 1.56)	2.20 (3.09 - 1.93)	2.14 (3.01 - 1.66)	0.936	ƞ^2^ = 0.001
TLM (kg)	19.09 (22.6 - 15.19)	18.78 (25.17 - 17.1)	19.13 (24.93 - 15.32)	0.501	ƞ^2^ = 0.018
LLM-R (kg)	6.51 (8.05 - 4.72)	6.04 (8.93 - 4.97)	6.05 (6.9 - 4.91)	0.452	ƞ^2^ = 0.006
LLM-L (kg)	6.58 (8.39 - 4.66)	6.18 (8.93 - 4.92)	5.90 (7.15 - 4.83)	0.521	ƞ^2^ = 0.006
SLM (kg)	40.90 (49.6 - 31.4)	38.80 (52.6 - 35.4)	40.88 (48.6 - 33.1)	0.767	ƞ^2^ = 0.003
FFM (kg)	43.40 (52.9 - 3.3)	41.30 (55.9 - 37.6)	43.44 (51.6 - 35.2)	0.767	ƞ^2^ = 0.003
CS (times/30s)^***^	20.00 (30 – 16 )^***^	21.00 (25 – 15 )^***^	15.00 (22 – 14 )^***^	<0.001	ƞ^2^ = 0.438
TB (s)^**^	4.52 (5.72 - 2.97)^*^	4.59 (9.38 - 2.43)^*^	5.42 (9.8 - 4.05)^*^	0.004	ƞ^2^ = 0.152
TUG (s)^***^	5.78 ± 0.79^***^	5.47 ± 0.59^***^	6.58 ± 0.5^***^	<0.001	ƞ^2^ = 0.363
AC-R (times/30s)^**^	19.00 (29 –15 )	21.00 (26 –16 )^**^	17.00 (21 –11 )^**^	0.003	ƞ^2^ = 0.165
AC-L (times/30s)	19.00 (31 – 15)	21.00 (27 – 15 )	18.00 (25 – 8 )	0.083	ƞ^2^ = 0.027
KES (Nm)	105.50 (170 - 83.3)	108.15 (164.9 - 74.9)	102.98 (119.9 - 61.4)	0.282	ƞ^2^ = 0.012
KFS (Nm)^***^	45.1 (63.3 - 34.4)^***^	45.65 (56.2 - 41.7)^***^	41.3 (48.4 - 33.9)^***^	<0.001	ƞ^2^ = 0.207
**Other**	**HIIT NW (n = 23)**	**ST (n = 24)**	**CG (n = 24)**		
Crea (mg/dl)	0.74 (1.1 - 0.65)	0.77 (1.5 - 0.6)	0.71 (0.92 - 0.62)	0.239	ƞ^2^ = 0.039
CK (u/l)	102.00 (376 − 40)	92.50 (238 − 50)	91.50 (136 − 52)	0.587	ƞ^2^ = 0.013
BMI (kg/m^2^)	26.45 ± 3.56	25.92 ± 3.64	28.03 ± 4.54	0.08	ƞ^2^ = 0.037
UAC-R (cm)	31.14 ± 2.56	30.92 ± 2.62	32.37 ± 3.42	0.19	ƞ^2^ = 0.037
UAC-L (cm)	31.08 ± 2.59	30.91 ± 2.61	32.32 ± 3.42	0.2	ƞ^2^ = 0.036
ULI-1 (kg)^**^	0.4 ± 0.08	0.43 ± 0.06^**^	0.36 ± 0.07^**^	0.01	ƞ^2^ = 0.124
ULI-2 (kg/m^2^)^*^	1.02 ± 0.21	1.1 ± 0.2^**^	0.94 ± 0.19^**^	0.03	ƞ^2^ = 0.099
LLI-1 (Nm/kg)	1.62 ± 0.39	1.65 ± 0.36	1.44 ± 0.35	0.09	ƞ^2^ = 0.034
LLI-2 (Nm/kg)^**^	0.65 ± 0.14	0.7 ± 0.14^**^	0.58 ± 0.11^**^	0.01	ƞ^2^ = 0.124

Note: SMM: Skeletal muscle mass; HS-R: Hand strength-right; HS-L: Hand strength-left; GS: Gait speed; SMI: Skeletal muscle index; BFM: Body fat mass; ALM-R: Arm lean mass-right; ALM-L: Arm lean mass-left; TLM: Trunk lean mass; LLM-R: Leg lean mass-right; LLM-L: Leg lean mass-left; SLM: Skeletal lean mass; FFM: Fat free mass; CS: Chair stand; TB: Tandem Balance; TUG: “Time up and go” test; AC-R: Armcurl-right; AC-L: Armcurl-left; KES: Knee extensor strength; KFS: Knee flexor strength; Crea: Creatinine; CK: Creatine kinase; BMI: Body mass index; UAC-R: Upper arm circumference-right; UAC-L: Upper arm circumference-left; ULI-1: Upper limbs strength index 1; ULI-2: Upper limbs strength index 2; LLI-1: Lower limb strength index 1; LLI-2: Lower limb strength index 2. Numbers connected by “±” are the mean and standard deviation, numbers before “-” are the median, and numbers connected by “-” are the maximum and minimum values; ^*^: p < 0.05, ^**^: p < 0.01, ^***^: p < 0.001. 95% confidence intervals were used for all data expressed as means and standard deviations. The effect size was represented by the value of eta^2^ (effect size criteria: small, ƞ^2^ = 0.01; Medium, ƞ^2^ = 0.06; Large, ƞ^2^ = 0.14

**Fig 3:**
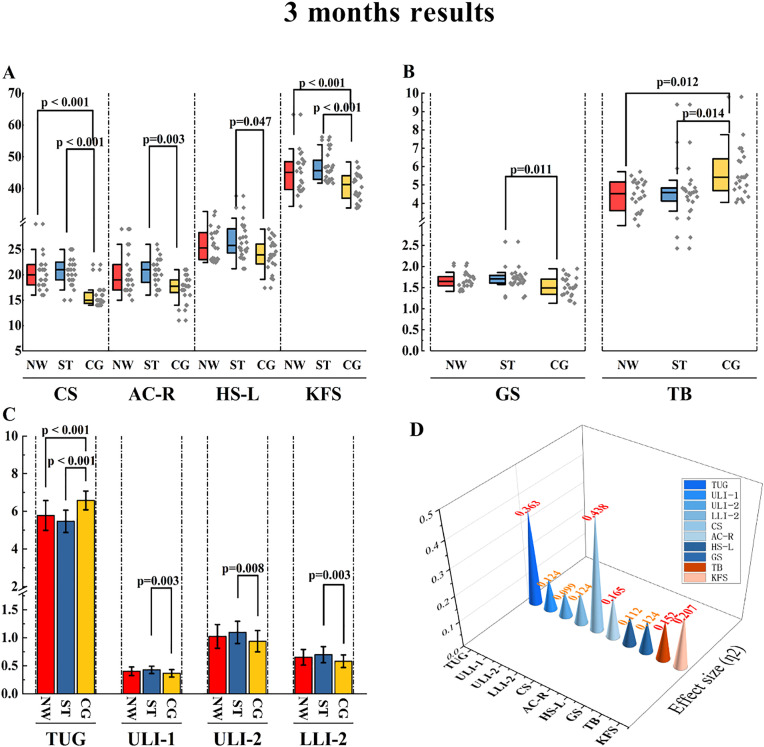
Comparison of data after the experiment. Note: Box plot A and B represent skewed distribution data, the center line of the box represents the median, and the gray prism represents the distribution position of each value. Bar C represents the normally distributed data, with the mean at the top of the bar and the black error bar showing the standard deviation. The 3D cone represents the effect size, with specific numbers at the top, red fonts for large effect sizes, and orange fonts for medium effect sizes. CS: Chair stand; AC-R: Armcurl-right; HS-L: Hand strength-left; KFS: Knee flexor strength; GS: Gait speed; TB: Tandem Balance; TUG: “Time up and go” test; ULI-1: Upper limbs strength index 1; ULI-2: Upper limbs strength index 2; LLI-2: Lower limb strength index 2.

After the experiment, Several outcome measures demonstrated statistically significant differences between the two experimental groups, as well as between each experimental group and the control group. Primary outcome indicators: Left hand grip strength (HS-L): ST group was significantly higher than control group (ST: 25.8 kg, CG: 23.92 kg, p = 0.047, ƞ^2 ^= 0.112, as shown in Fig 3A, 3D). It is worth mentioning that 2 of the 3 left-handed participants were in the ST group and 1 in the CG group. The proportion of left-handed persons in the ST group was 8.33%, which may be used as an explanation for the significant improvement in the ST group. Gait speed (GS): ST group was significantly higher than control group (ST: 1.71 m/s, CG: 1.49 m/s, p = 0.011, ƞ^2 ^= 0.124, as shown in Fig 3B, 3D).

Secondary outcome indicators: Chair Stand (CS): HIIT NW group and ST group were significantly higher than the control group (HIIT NW: 20, ST: 21, CG: 15, p < 0.001, ƞ^2^ = 0.438, as shown in Fig 3A, 3D). Right Arm Curl (AC-R): ST group was significantly higher than control group (ST: 21, CG: 17.74, p = 0.003, ƞ^2^ = 0.165, as shown in Fig 3A, 3D). Knee flexor strength (KFS) in HIIT NW and ST groups was significantly higher than that in control group (HIIT NW: 45.5.1 Nm, ST: 45.65 Nm, CG: 41.3 Nm, p < 0.001, ƞ^2^ = 0.207, as shown in Fig 3A, 3D). Tandem Balance (TB): HIIT NW group and ST group were significantly better than the control group (HIIT NW: 4.52 s, ST: 4.59 s, CG: 5.42 s, p = 0.012, p = 0.014, respectively, ƞ^2 ^= 0.152, as shown in Fig 3B, 3D). Time Up and Go (TUG): The HIIT NW group and the ST group were significantly better than the control group (HIIT NW: 5.78 ± 0.79s, ST: 5.47 ± 0.59s, CG: 6.58 ± 0.5s, p < 0.001, ƞ^2 ^= 0.363, as shown in Fig 3C, 3D).

Other outcome indicators: Upper limbs strength index 1 (ULI-1): ST group was significantly higher than control group (ST: 0.43 ± 0.06 kg, CG: 0.36 ± 0.07 kg, p = 0.003, ƞ^2^ = 0.124, as shown in Fig 3C, 3D). Upper limbs strength index 2 (ULI-2): ST group was significantly higher than control group (ST: 1.1 ± 0.2 kg/m^2^, CG: 0.94 ± 0.19 kg/m^2^, p = 0.008, ƞ^2^ = 0.099, as shown in Fig 3C, 3D). Lower limb strength index 2 (LLI-2): ST group was significantly higher than control group (ST: 0.7 ± 0.14 Nm/kg, CG: 0.58 ± 0.11 Nm/kg, p = 0.003, ƞ^2^ = 0.124, as shown in Fig 3C, 3D).

### 3.3. Intra-group responses to activities

The results for each of the three groups are shown in Table 3.

**Table 3:** Comparison before and after the experiment in each group.

	Outcomes	Before and after experiment		P
**HIIT NW (n = 23)**	**Primary**	**Pre**	**Post**	
	SMM (kg)^***^	22.86 ± 2.45	23.95 ± 2.05^***^	<0.001
	SMI (kg/m2)^***^	6.61 ± 0.56	6.82 ± 0.53^***^	<0.001
	HS-R (kg)^***^	24.4(32.6 - 20.1)	25.3(35.2 − 23)^***^	<0.001
	HS-L (kg)^**^	24.1(30.1 - 20.1)	25.3(32.8 - 22.4)^**^	0.005
	GS (m/s)^***^	1.53 ± 0.21	1.66 ± 0.18^***^	<0.001
	**Secondary**	**Pre**	**Post**	
	BFM (kg)	25.56 ± 6.98	24.53 ± 7.2	0.052
	SLM (kg)	40.5 ± 4.3	40.45 ± 4.32	0.877
	FFM (kg)	43.07 ± 4.59	43.01 ± 4.62	0.855
	ALM-R (kg)^**^	2.16 ± 0.3	2.25 ± 0.27^**^	0.002
	ALM-L (kg)^**^	2.13 ± 0.33	2.22 ± 0.31^**^	0.002
	TLM (kg)	19.43 ± 1.9	19.07 ± 1.97	0.079
	LLM-R (kg)^***^	6.32 ± 0.92	6.48 ± 0.91^***^	<0.001
	LLM-L (kg)^***^	6.37 ± 0.96	6.55 ± 0.96^***^	<0.001
	KES (Nm)^***^	99.7(136.3 - 55.7)	105.5(170 - 83.3)^***^	0.001
	KFS (Nm)^***^	38.86 ± 4.11	44.89 ± 6.39^***^	<0.001
	CS (times/30s)^***^	16(21 – 11 )	20(30 – 16 )^***^	<0.001
	TB (s)^*^	4.99(7.95 - 4.09)	4.52(5.72 - 2.97)^*^	0.017
	TUG (s)^**^	5.97(8.82 - 5.13)	5.85(7.19 - 4.3)^**^	0.003
	AC-R (times/30s)^***^	16(24 – 13)	19(29 – 15 )^***^	<0.001
	AC-L (times/30s)^***^	17(23 – 12)	19(31 – 15 )^***^	<0.001
	**Other**	**Pre**	**Post**	
	BMI (kg/m^2^)	26.87 ± 3.36	26.45 ± 3.56	0.137
	UAC-R (cm)	31.56 ± 2.72	31.14 ± 2.56	0.077
	UAC-L (cm)	31.46 ± 2.7	31.08 ± 2.59	0.09
	Crea (mg/dl)	0.77 (1.02 - 0.62)	0.74 (1.1 - 0.65)	0.649
	CK (u/l)^**^	73(263 − 38)	102(376 − 40)^**^	0.006
	ULI-1 (kg)^*^	0.37(0.63 - 0.28)	0.4(0.62 - 0.26)^*^	0.019
	ULI-2 (kg/m^2^)^*^	0.94(1.65 - 0.71)	0.97(1.63 - 0.73)^*^	0.021
	LLI-1 (Nm/kg)^**^	1.49 ± 0.35	1.62 ± 0.39^**^	0.005
	LLI-2 (Nm/kg)^**^	0.59 ± 0.1	0.65 ± 0.14^**^	0.002
**ST** **(n = 24)**	**Primary**	**Pre**	**Post**	
	SMI (kg/m^2^)	6.49 (8.67 - 5.73)	6.57 (8.64 - 5.99)	0.072
	SMM (kg)^***^	22.42(31.03 - 19.57)	23.72(30.74 - 21.77)^***^	<0.001
	HS-R (kg)^***^	25.2(34.3 - 17.7)	27.25(43.5 - 22.3)^***^	<0.001
	HS-L (kg)^***^	24.68 ± 3.96	26.71 ± 3.86^***^	0.001
	GS (m/s)^***^	1.57(1.76 - 1.27)	1.71(2.59 - 1.26)^***^	0.001
	**Secondary**	**Pre**	**Post**	
	SLM (kg)	39.3 (53 - 34.6)	38.8 (52.6 - 35.4)	0.558
	FFM (kg)	41.7 (56.3 - 36.8)	41.3 (55.9 - 37.6)	0.493
	BFM (kg)^***^	25 ± 7.8	23.4 ± 7.44^***^	<0.001
	ALM-R (kg)	2.16 (3.22 - 1.62)	2.23 (3.16 - 2.01)	0.485
	ALM-L (kg)	2.1 (3.23 - 1.65)	2.2 (3.09 - 1.93)	0.753
	LLM-R (kg)	6.26 ± 0.88	6.31 ± 0.93	0.174
	LLM-L (kg)	6.31 ± 0.88	6.38 ± 0.94	0.105
	TLM (kg)	18.82 (25.72 - 16.43)	18.78 (25.17 - 17.1)	0.449
	KES (Nm)	102.13 ± 17.87	109.01 ± 21.55	0.087
	KFS (Nm)^***^	42.32(49.4 - 34.1)	45.65(56.2 - 41.7)^***^	<0.001
	CS (times/30s)^***^	16.42 ± 2.34	20.83 ± 2.66^***^	<0.001
	TB (s)^*^	5.18(9.11 - 3.67)	4.59(9.38 - 2.43)^*^	0.021
	TUG (s)^***^	6.16 ± 0.64	5.47 ± 0.59^***^	<0.001
	AC-R (times/30s)^***^	17.5(21 – 14 )	21(26 – 16 )^***^	<0.001
	AC-L (times/30s)^***^	18.17 ± 2.14	20.42 ± 2.87^***^	0.001
	**Other**	**Pre**	**Post**	
	BMI (kg/m^2^)^**^	26.51 ± 4.01	25.92 ± 3.64^**^	0.005
	ULC-R (cm)^*^	31.32 ± 2.86	30.92 ± 2.62^*^	0.034
	UAC-L (cm)	31.23 ± 2.87	30.91 ± 2.61	0.072
	ULI-1 (kg)^***^	0.39 ± 0.06	0.43 ± 0.06	<0.001
	ULI-2 (kg/m^2^)^***^	1.01 ± 0.18	1.1 ± 0.2	<0.001
	LLI-1 (Nm/kg)	1.54 ± 0.33	1.65 ± 0.36	0.064
	LLI-2 (Nm/kg)^***^	0.63 ± 0.12	0.7 ± 0.14^***^	0.001
	CK (u/l)^*^	85.5(160 − 41)	92.5(238 − 50)^*^	0.016
	Crea (mg/dl)	0.76 (1.01 - 0.6)	0.77 (1.5 - 0.6)	0.678
**CG** **(n = 24)**	**Primary**	**Pre**	**Post**	
	SMM (kg)	23.39 ± 2.66	22.89 ± 1.9	0.172
	HS-R (kg)	26.15 ± 4.37	25.13 ± 3.38	0.199
	HS-L (kg)	24.81 ± 3.76	23.92 ± 3.05	0.223
	GS (m/s)	1.58 ± 0.24	1.52 ± 0.23	0.14
	SMI (kg/m^2^)	6.69 ± 0.56	6.61 ± 0.54	0.455
	**Secondary**	**Pre**	**Post**	
	BFM (kg)	27.71 ± 9.99	28.55 ± 9.61	0.403
	SLM (kg)	40.82 ± 4.25	40.88 ± 4.03	0.889
	FFM (kg)	43.36 ± 4.49	43.44 ± 4.29	0.882
	ALM-R (kg)	2.33 ± 0.35	2.29 ± 0.34	0.194
	ALM-L (kg)	2.3 ± 0.34	2.24 ± 0.33	0.098
	TLM (kg)	20.02 ± 2.26	19.73 ± 2.18	0.162
	LLM-R (kg)	6.17 (7.76 - 4.74)	6.45 (6.9 - 4.91)	0.511
	LLM-L (kg)	6.26 (7.93 - 4.64)	6.5 (7.15 - 4.83)	0.466
	AC-R (times/30s)	17 ± 2.91	17 ± 2.55	0.302
	AC-L (times/30s)	18 (23 – 10)	18 (25 – 6)	0.221
	CS (times/30s)	17 (22 – 15 )	15 (22 – 14)	0.073
	TUG (s)	6.31 (8.04 - 5.09)	6.46 (8.11 - 6.09)	0.548
	TB (s)	5.28 (7.95 - 3.25)	5.42 (9.8 - 4.05)	0.989
	KES (Nm)	99.08 (152.1 - 44.9)	102.98 (119.9 - 61.4)	0.886
	KFS (Nm)	41.46 ± 8.6	40.56 ± 4.5	0.644
	**Other**	**Pre**	**Post**	
	BMI (kg/m^2^)	28.01 ± 4.91	28.03 ± 4.54	0.96
	UAC-R (cm)	32.55 ± 3.86	32.37 ± 3.42	0.595
	UAC-L (cm)	32.44 ± 3.92	32.32 ± 3.42	0.734
	ULI-1 (kg)	0.38 ± 0.08	0.36 ± 0.07	0.148
	ULI-2 (kg/m^2^)	0.99 ± 0.25	0.94 ± 0.19	0.138
	LLI-1 (Nm/kg)	1.44 ± 0.43	1.44 ± 0.35	0.97
	LLI-2 (Nm/kg)	0.59 ± 0.15	0.58 ± 0.11	0.677
	CK (u/l)	85.88 ± 31.97	86.93 ± 32.23	0.865
	Crea (mg/dl)	0.73 (0.95 - 0.65)	0.71 (0.92 - 0.62)	0.696

Note: SMM: Skeletal muscle mass; HS-R: Hand strength-right; HS-L: Hand strength-left; GS: Gait speed; SMI: Skeletal muscle index; BFM: Body fat mass; ALM-R: Arm lean mass-right; ALM-L: Arm lean mass-left; TLM: Trunk lean mass; LLM-R: Leg lean mass-right; LLM-L: Leg lean mass-left; SLM: Skeletal lean mass; FFM: Fat free mass; CS: Chair stand; TB: Tandem Balance; TUG: “Time up and go” test; AC-R: Armcurl-right; AC-L: Armcurl-left; KES: Knee extensor strength; KFS: Knee flexor strength; Crea: Creatinine; CK: Creatine kinase; BMI: Body mass index; UAC-R: Upper arm circumference-right; UAC-L: Upper arm circumference-left; ULI-1: Upper limbs strength index 1; ULI-2: Upper limbs strength index 2; LLI-1: Lower limb strength index 1; LLI-2: Lower limb strength index 2. Numbers connected by “±” are the mean and standard deviation, numbers before “-” are the median, and numbers connected by “-” are the maximum and minimum values; ^*^: p < 0.05, ^**^: p < 0.01, ^***^: p < 0.001. 95% confidence intervals were used for all data expressed as means and standard deviations.

#### 3.3.1. Comparison within the HIIT NW group.

**Fig 4:**
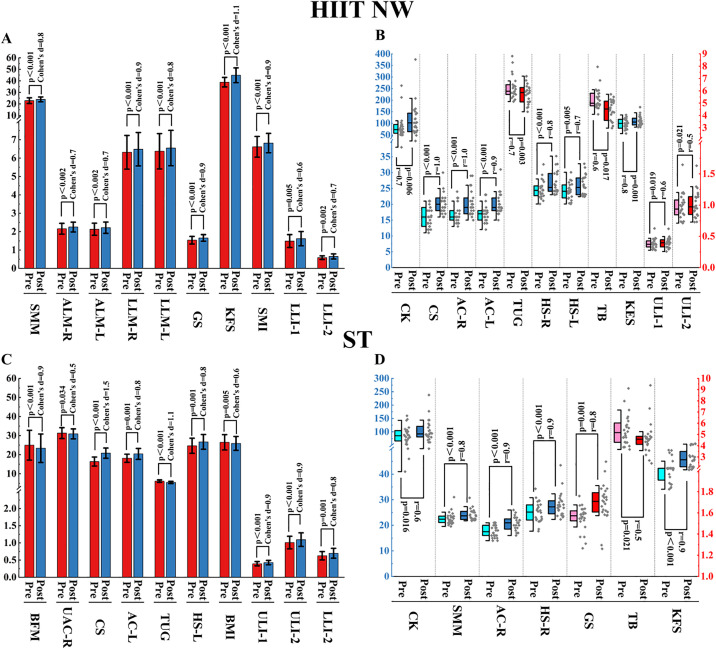
Comparison within groups. Note: HIIT NW: NW training based on high-intensity interval training, ST: Strength Training, p: p-value, r: rank biserial correlation coefficient. The blue and red boxes correspond to the blue and red y-axes respectively. SMM: Skeletal muscle mass; ALM-R: Arm lean mass-right; ALM-L: Arm lean mass-left; LLM-R: Leg lean mass-right; LLM-L: Leg lean mass-left; GS: Gait speed; KFS: Knee flexor strength; SMI: Skeletal muscle index; LLI-1: Lower limb strength index 1; LLI-2: Lower limb strength index 2; CK: Creatine kinase; CS: Chair stand; AC-R: Armcurl-right; AC-L: Armcurl-left; TUG: “Time up and go” test; HS-R: Hand strength-right; HS-L: Hand strength-left; TB: Tandem Balance; KES: Knee extensor strength; ULI-1: Upper limbs strength index 1; ULI-2: Upper limbs strength index 2; BFM: Body fat mass; UAC-R: Upper arm circumference-right; BMI: Body mass index.

In this study, all outcome indicators with significant differences are listed in Fig 4. After the HIIT NW programme, of the primary indicators, skeletal muscle mass (SMM) was significantly increased (Pre: 22.86 ± 2.45, Post: 23.95 ± 2.05, p < 0.001, Cohen’s d = 0.8) as was gait speed (GS) (Pre: 1.53 ± 0.21 s/m, Post: 1.66 ± 0.18 s/m, p < 0.001, Cohen’s d = 0.9), skeletal muscle index (SMI) (Pre: 6.61 ± 0.56, Post: 6.82 ± 0.53, p < 0.001, Cohen’s d = 0.9), strength of the right hand (HS-R) (Pre: 24.4 kg, Post: 25.3 kg, p < 0.001, r = 0.8), strength of the left hand (HS-L) (Pre: 24.1 kg, Post: 25.3 kg, p = 0.005, r = 0.7).

After the HIIT NW programme, of the secondary indicators, lean mass of both arms (ALM-R, ALM-L) was significantly increased (Pre: 2.16 ± 0.3, Post: 2.25 ± 0.27; Pre: 2.13 ± 0.33, Post: 2.22 ± 0.31, p = 0.002, Cohen’s d = 0.7, respectively) as was lean mass of both legs (LLM-R, LLM-L) (Pre: 6.32 ± 0.92, Post: 6.48 ± 0.91; Pre: 6.37 ± 0.96, Post: 6.55 ± 0.96, p < 0.001, Cohen’s d = 0.9, 0.8, respectively), the strength of knee flexors and extensors (KFS, KES) (Pre: 38.86 ± 4.11 Nm, Post: 44.89 ± 6.39 Nm, p < 0.001, Cohen’s d = 1.1, Pre: 99.7 Nm, Post: 105.5 Nm, p = 0.001, r = 0.8, respectively), chair stand (CS) (Pre: 16, Post: 20, p < 0.001, r = 1), the number of curls of both arms (AC-R, AC-L) (Pre: 16, Post: 19; Pre: 17, Post: 19, p < 0.001, r = 1.0, 0.9, respectively), both walking tests (TUG, TB) (Pre: 5.97 s, Post: 5.58 s, p = 0.003, r = 0.7; Pre: 4.99s, Post: 4.52s, p = 0.017, r = 0.6, respectively).

After the HIIT NW programme, of the other indicators, the strength index of the two lower limbs (LLI-1, LLI-2) was significantly increased (Pre: 1.49 ± 0.35 Nm/kg, Post: 1.62 ± 0.39 Nm/kg, p = 0.005, Cohen’s d = 6, Pre: 0.59 ± 0.1 Nm/kg, Post: 0.65 ± 0.14 Nm/kg, p = 0.002, Cohen’s d = 0.7, respectively) as was both upper body strength indexes (ULI-1, ULI-2) (Pre: 0.37 kg, Post: 0.4 kg, p = 0.019, r = 0.6, Pre: 0.94 kg/m^2^, Post: 0.97 kg/m^2^, p = 0.021, r = 0.5, respectively), creatine kinase (CK) (Pre: 73, Post: 102, p = 0.006, r = 0.7).

#### 3.3.2. Comparison within the ST group.

In this study, all outcome indicators with significant differences are listed in Fig 4. After the ST programme, of the primary indicators, the grip strength of both hands (HS-L, HS-R) was significantly increased (Pre: 24.68 ± 3.96 kg, Post: 26.71 ± 3.86 kg, p = 0.001, Cohen’s d = 0.8; Pre: 25.2 kg, Post: 27.25 kg, p < 0.001, r = 0.9, respectively) as was skeletal muscle mass (SMM) (Pre: 22.42, Post: 23.72, p < 0.001, r = 0.8) gait speed (GS) (Pre: 1.57 m/s, Post: 1.71 m/s, p = 0.001, r = 0.8).

After the ST programme, of the secondary indicators, body fat mass and body mass index (BFM, BMI) was significantly decreased (Pre: 25 ± 7.8, Post: 23.4 ± 7.44, p < 0.001, Cohen’s d = 0.9; Pre: 26.51 ± 4.01, Post: 25.92 ± 3.64, p = 0.005, Cohen’s d = 0.6, respectively). The chair stand (CS) was significantly increased (Pre: 16 ± 2.34, Post: 21 ± 2.66, p < 0.001, Cohen’s d = 1.5) as was the number of arm curl (AC-L, AC-R) (Pre: 18 ± 2.14, Post: 20 ± 2.87, p = 0.001, Cohen’s d = 0.8; Pre: 18, Post: 21, p < 0.001, r = 0.9, respectively), two tests on walking (TUG, TB) (Pre: 6.16 ± 0.64 s, Post: 5.47 ± 0.59 s, p < 0.001, Cohen’s d = 1.1; Pre: 5.18 s, Post: 4.59 s, p = 0.021, r = 0.5, respectively), knee flexor strength (KFS) (Pre: 42.32 Nm, Post: 45.65 Nm, p < 0.001, r = 0.9).

After the ST programme, of the other indicators, the right upper arm circumference (UAC-R) was significantly decreased (Pre: 31.32 ± 2.86 cm, Post: 30.92 ± 2.62 cm, p = 0.034, Cohen’s d = 0.5). the strength indexes of the three limbs (ULI-1, ULI-2, LLI-2) was significantly increased (Pre: 0.39 ± 0.06, Post: 0.43 ± 0.06, p < 0.001, Cohen’s d = 0.9; Pre: 1.01 ± 0.18, Post: 1.1 ± 0.2, p < 0.001, Cohen’s d = 0.9; Pre: 0.63 ± 0.12, Post: 0.7 ± 0.4, p = 0.001, Cohen’s d = 0.8, respectively), creatine kinase (CK) (Pre: 85.5, Post: 92.5, p = 0.016, r = 0.6).

### 3.4. Correlations between body composition and physical function and parameters related to weekly walking

**Fig 5:**
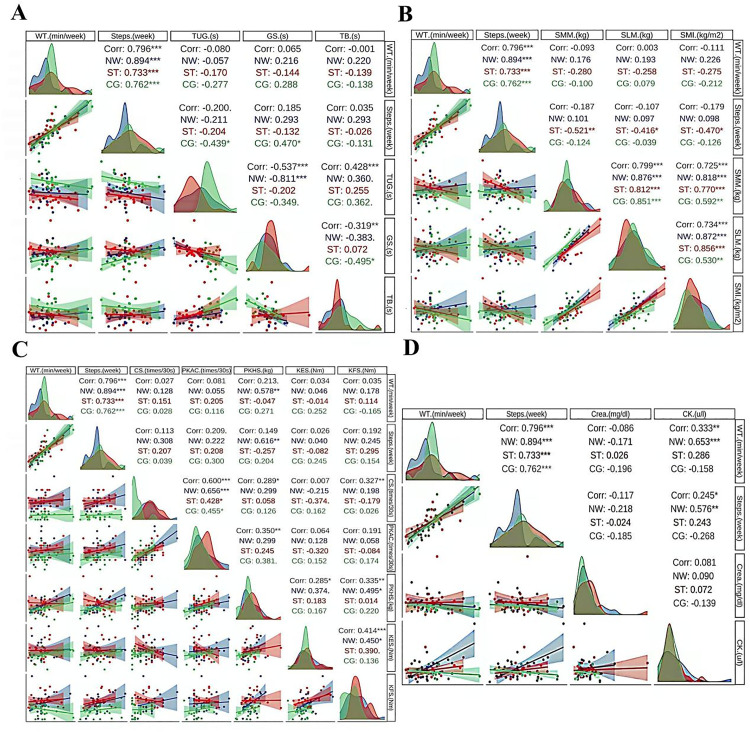
Correlation diagram of each parameter in each group. Note: WT: Walking time; PKAC: peak of arm curl; PKHS: peak of hand strength. other abbreviations can be found in the outcome section. Corr: For any two variables, the closer the correlation coefficient is to 1, or −1 the stronger the relationship either positive or negative. *: Significance (* = 0.05, ** = 0.01, *** = 0.001). The curve graph is used to show the distribution of the data, and the scatter plot is used to show the degree of linear fit of the data, including prediction bands for each group of data.

This study conducted correlation analysis on parameters related to body composition (SMM, SML, SMI), physical function associated with walking (TUG, GS, TB), physical functions related to strength (CS, PKAC, PKHS, KES, KFS), blood index (Crea and CK), and weekly walking (Time and Steps) in each group. It should be noted that weekly walking time and number of steps were not included in the intervention period, as shown in Fig 5.

Some of the measures associated with walking (TUG, GS, and TB) were not significantly associated with walking time and step count overall. The faster GS, the less TUG and TB were used (r: −0.573, p < 0.001, r: −0.319, p < 0.01, respectively). The relationship between GS and TUG is most significant in HIIT NW group, while the relationship between GS and TB is more significant in CG group, as shown in Fig 5A.

On the whole, there was no significant correlation between the body composition indices (SMM, SLM, SMI), which reflected the level of skeletal muscle, and the weekly walking time and steps (r: −0.093, 0.003, −0.1111, p > 0.05; r: −0.187, −0.017, −0.179, p > 0.05, respectively). The body composition index of ST group was negatively correlated with the number of steps in different degrees (r: −0.521, p < 0.01, r: −0.416, p < 0.05, Corr: −0.47, p < 0.05, respectively). In addition, there was a significant positive correlation between the indexes of body composition, as shown in Fig 5B.

Summarizing, some physical function indicators (CS, PKAC, PKHS, KES, KFS) reflecting skeletal muscle level were not significantly correlated with weekly walking time and steps (r: 0.027, 0.081, 0.213, 0.034, 0.035; r: 0.113, 0.209, 0.149, 0.026, 0.192, p > 0.05, respectively. In addition, CS was positively correlated with PKAC, PKHS and KFS (r: 0.6, p < 0.001; r: 0.289, p < 0.05; Corr: 0.327, p < 0.01, respectively. There was significant positive correlation between PKAC and PKHS (r: 0.35, p < 0.01). PKHS was positively correlated with KES and KFS (r: 0.285, p < 0.05; r: 0.335, p < 0.01, respectively). KES and KFS were positively correlated (r: 0.414, p < 0.001), as shown in Fig 5C.

Summarizing, there was no significant correlation between blood index (Crea) and walking time and step number (r: −0.086, −0.117, p > 0.05), but there was a significant positive correlation between the other blood index (CK) and walking time (r: 0.333, p < 0.01; r: 0.245, p < 0.05, respectively), and the HIIT NW group was the most significant (r: 0.653, p < 0.001; r: 0.576, p < 0.01 respectively). In addition, there is no significant correlation between Crea and CK, as shown in Fig 5D.

## 4. Discussion

In comparison to the control group, both the HIIT NW and ST groups shown substantial enhancements in four physical function metrics (CS, KFS, TUG, and TB). The ST group demonstrated substantial enhancements in the major outcome measure (GS). In the intra-group comparisons, the HIIT NW group shown substantial enhancements in SMI, SMM, GS, and HS. The ST group exhibited notable enhancements in SMM, GS, and HS. The results indicate that both therapies were successful in mitigating sarcopenia. The HIIT NW intervention primarily enhanced lower limb strength, while the ST intervention predominantly improved upper body strength.

Similar studies have shown that short-term NW and HIIT intervention patterns can improve skeletal muscle mass in older adults [53]. This study observed a notable increase in limb lean mass among patients participating in the HIIT-based Nordic walking intervention, resulting in a considerable enhancement in SMI, likely due to the increased resistance provided to the upper limbs throughout the walking regimen. Marciniak et al. concur with our position [54]. In their trial, individuals in the HIIT NW group reported a 12 percent improvement in an upper body strength (AC) test, compared to 18.75 percent in this study under identical conditions. Youssef et al. discovered that HIIT training improved participants’ gait speed when evaluated over a length of 4 meters, resulting in an 8.03% improvement, whereas this study tested over a length of 6 meters and found an 8.5% improvement. HIIT did not significantly enhance HS levels (3.38% rise, p = 0.46), whereas this study found a 3.69% increase, p < 0.001. This appears to indicate that NW paired with HIIT training has a greater effect on HS [55]. It needs to be clarified that the use of poles in HIIT NW training needs to be emphasized by participants, which is likely to be the key to improving upper limb strength, especially grip strength. The HIIT-based NW training mode appears to be more effective for increasing lower limb strength. Regular ST is excellent for developing upper-body strength. This could be because the HIIT-based NW training mode is still primarily focused on developing lower limb strength; faster cross-country walking is an important aspect in improving lower limb strength, but high-intensity interval training should not be overlooked. It is worth noting that, while the improvement in upper body strength in the HIIT NW group is not as significant as that in the ST group, it does not imply that there is no improvement, which may explain why the SMI in the HIIT NW group is significantly higher. This study found that it enhanced overall limb strength, particularly in the lower limbs. The gain in upper body strength in the ST group could be attributed to the use of conventional strength training equipment and focused training routines (for example, weight-bearing arm curls can be used to improve bicep strength). On this basis, the ST group’s training regimen includes exercises for the pectoralis major, latissimus dorsi, and abdominal muscles, and the general gain in upper body strength appears to be natural. The ST group’s training regimen focuses on the entire body, while the HIIT NW group focuses on the lower limbs during the primary training stage. This may explain why the ST group’s lower limb strength improvement is lower than that of the inHIIT NW group. Nonetheless, the ST group maintains a tiny lead in the GS test. This study indicated that after 12 weeks, BFM, BMI, and UAC-R dropped significantly in the ST group, but not in the HIIT NW group. One study found that 8 weeks of HIIT NW training did not reduce BW and BMI [56]. This appears to imply that the intervention pattern in the ST group is more helpful for those who are at risk of both obesity and sarcopenia than for those who are only at risk of sarcopenia, as the former benefit more from body fat loss and muscle mass gain. Furthermore, lower limb muscle strength is one of the major variables in avoiding fall risk, and HIIT NW training patterns improved knee extensor strength compared to ST, as observed in a 3-week, 5-times-a-week clinical experiment [57]. Notably, the correlation test revealed a significant negative correlation between GS test scores (speed) and TUG test scores (time) in the HIIT NW group, indicating a possible relationship between the two, consistent with the rationale that increased speed correlates with shorter time over the same distance. As a result, the HIIT NW intervention appears to improve functional performance in walking more efficiently than ST.

It is widely recognized that for certain diseases, prevention supersedes intervention. Consequently, postmenopausal women susceptible to sarcopenia should persist with both the NW-based HIIT exercise regimen and the conventional ST regimen. This study presents evidence that the HIIT-based NW training regimen may enhance lower body strength more effectively than the typical ST regimen for upper body strength. Notably, the former did not decrease the body weight and body mass index of the subjects, whereas the latter did so significantly. This study posits that the NW training model, when integrated with HIIT for the first time, merits further investigation. It also recommends that future research should expand the sample size to encompass a more diverse population and extend the duration of the intervention. This study did not assess if the individuals’ metabolic rate, cardiorespiratory function, and daily activities were enhanced. This study suggests that this topic can be examined from multiple aspects, necessitating the incorporation of unique assessment indicators for comprehensive observation and evaluation.

## 5. Limitations

There are some limitations in this study. First, a large number of samples were excluded, resulting in a small sample size and skewed distribution of some data, limiting further analysis.

Second, the use of randomization based on geographical location also had some limitations. First, although the distances between the cities were relatively small, this method does not fully eliminate the risk of uneven distribution of other variables, such as education level or access to healthcare, which may differ between cities. Additionally, geographical location may introduce some biases related to differences in health habits among residents of different regions, which could affect the overall representativeness of the sample. Despite these limitations, the use of geographical location-based division allowed for controlling environmental variables, which increased the internal validity of the study by minimizing the risk of systematic differences between groups.It is important to note that the quasi-randomized experimental design and some ill-considered design may lead to a risk of bias in the results (e.g., a significant increase in left-hand grip strength in the HIIT NW and ST groups (p = 0.47), but ƞ^2^ is small, which may be a false positive). No Bonferroni correction was used in the mathematical analyses, so p values that were not used for multiple comparisons should be interpreted with caution. Data should be interpreted in light of the fact that adherence to the usual diet was not managed.

After that, there are some flaws in the design of this experiment. For example, participants were not assessed for physical strength, circadian rhythms were not considered, and nutritional intake was not controlled before testing. Adherence was not managed more rigorously during the study (e.g., external factors such as medication intake, nutrition supplementation, etc.).

Finally, the problems exposed by this short-term three-month experiment do not fully illustrate the problems that exist in the real world, and longer intervention cycles are needed to prove these problems.

## 6. Conclusion

In the limitations section, we describe the possible influence of quasi-randomized controlled trials on the results and the limitations of the study design itself.

Compared with the CG group, both the HIIT-based NW and ST intervention modes reduced the risk of sarcopenia in postmenopausal women, particularly with respect to SMM. The HIIT-based NW movement pattern is more conducive to improving lower limb strength. The regular ST exercise pattern is better for improving upper body strength. In the short term, the HIIT-based NW intervention model appears to be more beneficial to postmenopausal women with normal weight or BMI, helping them prevent sarcopenia. In the short term, the ST intervention model appears to be more beneficial for overweight postmenopausal women, helping them prevent muscle-slacking obesity.

The evidence and design ideas in the study may become the basis of reference for other researchers in the future (e.g., a randomized controlled trial with a larger sample size, longer duration, and more rigorous adherence management), and the questions raised in the study deserve more attention (e.g., incorporating HIIT NW and ST training into community exercise programs or prescribing similar exercise to patients in healthcare Settings).

## Supporting information

S1 File TREND Checklist. (DOC)

S2 File Data ste. (XLSX)

S3 File Information on the study. (PDF)

S4 File Informacja o badaniu. (PDF)

S5 File Information about the study. (DOC)

S6 File Research plan and informed consent. (PDF)

S7 File Research plan and informed consent-Translate. (PDF)

S8 File Plos-STUDY PROGRAM. (DOCX)
