# Supplementary material for: Effect of short-term exercise with different programs on prevention of sarcopenia in postmenopausal women: A Quasi-Randomized Controlled Trial
Source: PLoS One. 2025 Sep 30;20(9):e0333171. doi: 10.1371/journal.pone.0333171 (PMC12483237; doi:10.1371/journal.pone.0333171)
Supplement: S3 File — (PDF) [file pone.0333171.s003.pdf]

INFORMATION ON THE STUDY

Study assumptions:

Study/project topic: Physical activity and selected risk indicators for disability in older people.

Storm cel

1. To determine the effect of targeted physical activity on cognitive function and prevention of the risk of sarcopenia, overweight and falls in subjects.
2. To determine the effect of different training programs on pelvic floor muscle activity.
3. Determining the relationship between physical fitness and selected blood biomarkers in the elderly.
4. To determine the effect of physical activity on the gut microflora profile in subjects.

Organizer: Academy of Physical Education and Sport in Gdansk.

Miejsce realizacji badai: Akademia Wychowania Fizycznego i Sportu (AWFiS), ul. Kazimierza Gorskiego 1, Exercise Laboratory.

The studies are planned to be carried out in phases between 21 February 2022 and 15 June 2029.

Research methodology:

Test methods

1. To record the level of physical fitness and selected risk factors for sarcopenia, falls, exercise incontinence, obesity and cardiovascular diseases in the elderly.
   a. Arm and leg muscle strength: Hand Grip, Biodex
   b. Static and dynamic equilibrium (Zebris platform)
   c. Cognitive tests: (WST and Trail Making Test A, B)
   d. Measurement of body composition and selected anthropometric indicators (InBody 720 composition analyzer, tailored centimeter, goniometer, anthropometer)
   e. Functional fitness (Senior Fitness Test, Gait speed, Stand and Walk Test)

f. Aerobic capacity (Oxycon)

g. Footscan

h. Reaction speed and coordination (Blink)

2. Blood pressure measurement.
3. Study of pelvic floor muscle activity using surface electromyography in the direction of urinary incontinence.
4. Blood laboratory tests (baseline: morphology, lipid profile, glucose, keratin kinase, CRP protein, ALT, aspate, creatinine, albumin, uric acid, total protein, calcium, electrolytes).
5. Intestinal microflora analysis.

   Biological material (blood and faeces) will be frozen for further biochemical, proteomic and genetic testing.
6. Determination of selected blood biomarkers (Luminex)
7. Analysis of diet and eating habits (Nuvero programme).
8. Assessment of health behaviour, cognitive function and level of physical activity (International Physical Activity Questionnaire-short version, psychological tests).

Organisation of the study and information on exercise activities: Senior citizens will participate in the study twice, i.e. Before (baseline studies) and after the exercise programme (final studies). Health training and health education sessions will be delivered in stages between 21 February 2022 and 15 June 2029 by qualified instructors. Each phase of the project will include a different exercise programme (e.g. New Walking training, equivalent exercises, water gymnastics, resistance training and general gymnastics). Classes will be held 2-3 times a week at 45-60 minutes for a period of 10-22 weeks.

Place of classes: AWFIS or for outdoor classes olive forests.

I INFORMATION ABOUT THE STUDY MEDICINAL PRODUCT: Not applicable

Inclusion and exclusion criteria according to age and gender of the subjects:

Inclusion criteria:

• Women and men over 60 years of age who do not have contraindications to exercise.

• Consent to participate in the study and in the physical activity programme.

Exclusion criteria:

• Absence from classes for more than 3 weeks e.g. Caused by illness or departure.
• Withdrawal from participation in the project.

Place of additional tests, including laboratory tests:

All studies (including collection of biological material) will be performed in Akademii Wychowania Fizycznego i Sportu (AWFiS), ul. Kazimierza Gorskiego 1, Exercise Laboratory.

Information on the insurance of the OC of the site and the investigator: Not applicable

The collection of blood and its basic characteristics will be performed by the personnel of the SYNEVO medical laboratory in Gdansk with the applicable insurance (contracted services).
In addition, project participants will be asked to take out individual accident insurance (ACI) for the duration of the research and exercise.

Information about the centre with regard to the possibility of carrying out the programme, including the approval of the centre's management to carry out the study

Consent to carry out the above. Research in the AWFIS Physical Exercise Laboratory, provided by the coordinator of DS. Laboratoriów AWFiS prof. Dr. Hab. Stanislaw Sawczyn (approved in November 2021)
