# Supplementary material for: Effect of short-term exercise with different programs on prevention of sarcopenia in postmenopausal women: A Quasi-Randomized Controlled Trial
Source: PLoS One. 2025 Sep 30;20(9):e0333171. doi: 10.1371/journal.pone.0333171 (PMC12483237; doi:10.1371/journal.pone.0333171)
Supplement: S4 File — (PDF) [file pone.0333171.s004.pdf]

INFORMACJA O BADANIU

Założenia badania:

Temat badania/projektu: Aktywność fizyczna a wybrane wskaźniki ryzyka wystąpienia niesprawności u osób starszych.

Cel badań

1. Określenie wpływu ukierunkowanej aktywności fizycznej na funkcje poznawcze oraz profilaktykę ryzyka sarkopenii, nadwagi i upadków u badanych osób.
2. Określenie wpływu zróżnicowanych programów treningowych na aktywność mięśni dna miednicy.
3. Określenie związku sprawności fizycznej z wybranymi biomarkerami krwi u osób starszych.
4. Określenie wpływu aktywności fizycznej na profil mikroflory jelitowej u badanych osób.

Organizator: Akademii Wychowania Fizycznego i Sportu w Gdańsku.
Miejsce realizacji badań: Akademia Wychowania Fizycznego i Sportu (AWFiS), ul. Kazimierza Górskiego 1, Laboratorium Wysiłku Fizycznego.

Planuje się, że badania będą przeprowadzone etapami w okresie: 21 luty 2022 rok – 15 czerwiec 2029 rok.

Metodyka badań:

Metody badań

1. Rejestracja poziomu sprawności fizycznej oraz wybranych czynników ryzyka występowania niesprawności u osób starszych w kierunku sarkopenii, upadków, wysiłkowego nietrzymania moczu, otyłości oraz chorób układu krążenia.
   a. siła mięśni ramion i nóg: dynamometr ręczny (Hand Grip, Biodex)
   b. równowaga statyczna i dynamiczna (platforma Zebris)
   c. testy poznawcze: (WST i Trail Making Test A, B)
   d. pomiar składu ciała i wybranych wskaźników antropometrycznych (analizator składu InBody 720, centymetr krawiecki, goniometr, antropometr)
   e. sprawność funkcjonalna (Senior Fitness Test, Gait speed, Test “Wstań i idź”)

f. wydolność tlenowa (Oksykon)

g. chód (Footscan)

h. szybkość reakcji i koordynacja ruchowa (Blink)

2. Pomiar ciśnienia tętniczego krwi.
3. Badanie aktywności mięśni dna miednicy z wykorzystaniem elektromiografii powierzchniowej w kierunku nietrzymania moczu.
4. Analiza laboratoryjna krwi (oznaczenia podstawowe): morfologia, profil lipidowy, glukoza, kinaza keratynowa, białko CRP, ALT, AspAT, kreatynina, albumina, kwas moczowy, białko całkowite, wapń, elektrolity).
5. Analiza mikroflory jelitowej.
   Materiał biologiczny (krew i kał) będzie mrożony w celu przeprowadzenia dodatkowych oznaczeń biochemicznych, proteomicznych i genetycznych.
6. Oznaczenie wybranych biomarkerów krwi (Luminex).
7. Analiza diety i nawyków żywieniowych (program Nuvero).
8. Ocena zachowań zdrowotnych, funkcji poznawczych oraz poziomu aktywności fizycznej (Międzynarodowy Kwestionariusz Aktywności Fizycznej – wersja skrócona, testy psychologiczne).

Organizacja badań oraz informacja o zajęciach ruchowych: Seniorzy będą uczestniczyć w badaniach dwukrotnie tj. przed (badania początkowe) i po zakończeniu programu zajęć ruchowych (badania końcowe). Zajęcia w postaci treningu zdrowotnego i edukacji zdrowotnej będą realizowane etapami w okresie 21 luty 2022 rok – 15 czerwiec 2029 rok, przez wykwalifikowanych instruktorów. Każdy z etapów projektu będzie obejmował inny program zajęć ruchowych (np. trening New Walking, ćwiczenia równoważne, gimnastyka w wodzie, trening oporowy oraz gimnastyka ogólnorozwojowa). Zajęcia będą prowadzone 2-3 razy w tygodniu po 45-60 min przez okres 10-22 tygodni.

Miejsce zajęć: AWFiS lub w przypadku zajęć plenerowych lasy oliwskie.

Informacja o badanym leku: nie dotyczy

Kryteria włączenia i wyłączenia z uwzględnieniem wieku, płci badanych:

Kryteria włączenia:

- Kobiety i mężczyźni po 60 roku życia, nie posiadający przeciwwskazań do wysiłku fizycznego.

- Podpisanie zgody na udział w badaniach oraz w programie aktywności fizycznej.

Kryteria wyłączenia:

- Nieobecność na zajęciach powyżej 3 tygodni np. spowodowane chorobą lub wyjazdem.
- Rezygnacja z udziału w projekcie.

Miejsce wykonywania badań dodatkowych w tym laboratoryjnych:

Wszystkie badania (włącznie z pobraniem materiału biologicznego) wykonywane będą w Akademii Wychowania Fizycznego i Sportu (AWFiS), ul. Kazimierza Górskiego 1, Laboratorium Wysiłku Fizycznego.

Informacje dotyczące ubezpieczenia OC ośrodka i badacza: nie dotyczy

Pobranie krwi oraz podstawowe jej oznaczenia wykona personel Laboratorium Medycznego Synevo w Gdańsku posiadający stosowane ubezpieczenie (usługi zlecone).

Ponadto uczestnicy projektu zostaną poproszeni o wykupienie indywidualnego ubezpieczenia od następstw nieszczęśliwych wypadków (NNW) w okresie trwania badań i zajęć ruchowych.

Informacje dotyczące ośrodka pod kątem możliwości realizacji programu w tym zgoda dyrekcji ośrodka na przeprowadzenie badania

Zgodę na przeprowadzenie ww. badań w Laboratorium Wysiłku Fizycznego AWFiS, udzielił koordynator ds. Laboratoriów AWFiS prof. dr hab. Stanisław Sawczyn (zgoda została udzielona w listopadzie 2021 roku)
