# Supplementary material for: Effect of short-term exercise with different programs on prevention of sarcopenia in postmenopausal women: A Quasi-Randomized Controlled Trial
Source: PLoS One. 2025 Sep 30;20(9):e0333171. doi: 10.1371/journal.pone.0333171 (PMC12483237; doi:10.1371/journal.pone.0333171)
Supplement: S5 File — (DOC) [file pone.0333171.s005.doc]

**INFORMATION ABOUT THE STUDY**

**Topic:** Physical activity and selected risk indicators of disability in the elderly.

**Purpose of research**

1. Determination of the influence of directed physical activity on cognitive functions and prevention of the risk of sarcopenia, overweight and falls in the examined persons.

2. Determination of the influence of various training programs on the activity of the pelvic floor muscles.

3. Determining the relationship between physical fitness and selected blood biomarkers in the elderly.

Blood serum was labeled and frozen.

**Place of research:** Academy of Physical Education and Sport (AWFiS), ul. Kazimierza Górskiego 1, Laboratory of Physical Effort.

The research has been completed

**Methods**

1. Registration of the level of physical fitness and selected risk factors of disability in the elderly towards sarcopenia, falls, stress urinary incontinence, obesity and cardiovascular diseases.

a. arm and leg strength: hand dynamometer (Hand Grip, Biodex)

b. static and dynamic balance (Zebris platform)

c. cognitive tests: (WST and Trail Making Test A, B)

d. measurement of body composition and selected anthropometric indicators (InBody 720 composition analyzer, tailor's tape measure, goniometer, anthropometer)

e. functional fitness (Senior Fitness Test, Gait speed, "Get up and go" test)

f. aerobic capacity (Oksykon)

g. gait (Footscan)

h. reaction speed and motor coordination (Blink)

2. Measurement of blood pressure.

3. Study of pelvic floor muscle activity using surface electromyography for urinary incontinence.

4. Laboratory analysis of blood (basic determinations): morphology, lipid profile, glucose, keratin kinase, CRP protein, ALT, AST, creatinine, albumin, uric acid, total protein, calcium, electrolytes).

6. Determination of selected blood biomarkers (Luminex).

7. Analysis of diet and eating habits, diet logs (Nuvero program).

8. Assessment of health behavior, quality of life, cognitive functions and the level of physical activity (International Physical Activity Questionnaire - short version, psychological tests-COG).

**Organization of research and information about physical activities:** Seniors participated in three studies: two experimental groups (EG) participated in the first studies (stage A, November 2021). The second study (stage B, February 2022) took place after 12 weeks. Between the A-B tests, women did not participate in the classes at AWFiS. During this period (A-B), the activity of women was monitored (IPAQ, accelerometer). A new control group (CG) joined Test 2 (B). After study no.2, women from two EG groups started to implement health training: Nordic Walking - HIIT (EG1) and resistance training (EG2). Classes were conducted 3 times a week, 60 minutes each, for a period of 12 weeks. The CG group did not participate in the classes at AWFiS. In May 2022, there were final tests (stage C) for all groups.

**Inclusion criteria:**

• Women over 60 years of age, with no contraindications to exercise.

• Signing consent to participate in research and physical activity program.
