# Supplementary material for: Effect of short-term exercise with different programs on prevention of sarcopenia in postmenopausal women: A Quasi-Randomized Controlled Trial
Source: PLoS One. 2025 Sep 30;20(9):e0333171. doi: 10.1371/journal.pone.0333171 (PMC12483237; doi:10.1371/journal.pone.0333171)
Supplement: S6 File — (PDF) [file pone.0333171.s006.pdf]

INFORMACJA O BADANIU I PROGRAMIE ZAJĘĆ RUCHOWYCH ORAZ ŚWIADOMA ZGODA UCZESTNIKA

Założenia badania:

Temat badania/projektu: Aktywność fizyczna a wybrane wskaźniki ryzyka wystąpienia niesprawności u osób starszych.

Organizator: Akademii Wychowania Fizycznego i Sportu w Gdańsku. Kierownik badania/projektu – dr hab. Zbigniew Ossowski, prof. AWFiS,

Miejsce realizacji badań: Akademia Wychowania Fizycznego i Sportu (AWFiS), ul. Kazimierza Górskiego 1, Laboratorium Wysiłku Fizycznego.

Postępowanie badawcze:

Badania obejmują:

- ocenę sprawności fizycznej i wybranych wskaźników zdrowia tj. ocena zdolności motorycznych, wydolności tlenowej i czynników ryzyka upadków, badania antropometryczne i ciśnienia tętniczego, określenie progu bólowego, badanie zachowań zdrowotnych (w tym analizę diety) oraz funkcji poznawczych.
- badania laboratoryjne krwi: oznaczenia podstawowe (morfologia, profil lipidowy, glukoza, kinaza keratynowa, białko CRP, ALT, AspAT, kreatynina, albumina, kwas moczowy, białko całkowite, wapń, elektrolity).
- badania mikroflory jelitowej.

Oznakowane kodem próbki Pani/Pana materiału biologicznego (krew i kał) będą przechowywane w bardzo niskich temperaturach w pomieszczeniu, do którego dostęp mają tylko osoby upoważnione w celu przeprowadzenia dodatkowych ich oznaczeń biochemicznych, proteomicznych i genetycznych. Pani/Pana materiał biologiczny będzie przechowywany bezterminowo aż do momentu jego całkowitego wykorzystania. W przypadku stwierdzenia utraty przydatności materiału do badań naukowych lub wycofania przez Panią/Pana zgody na jego użycie, materiał zostanie zniszczony zgodnie z procedurami obowiązującymi w AWFiS w Gdańsku.

Zainteresowane Panie mogą ponadto wziąć udział w badaniu aktywności mięśni dna miednicy z wykorzystaniem elektromiografii powierzchniowej w kierunku nietrzymania moczu. Pani udział w badaniach będzie polegał na:

a. wypełnieniu tzw. metryczki badanych, zawierającej informacje o Pani wieku, masie ciała, problemach z zaburzeniami moczu w rodzinie itd.
b. wypełnieniu *Kwestionariusza wpływu nietrzymania moczu na życie codzienne* (*Incontinence Impact Questionnaire – IIQ)*; określi Pani czy i w jakich sytuacjach oraz w jakim stopniu doświadcza nietrzymania moczu.
c. poddaniu się ocenie funkcji mięśni dna miednicy przy zastosowaniu elektromiografii powierzchniowej wraz z zastosowaniem treningu metodą biofeedback. Ocena funkcji mięśni dna miednicy odbędzie się przy użyciu elektrody waginalnej, którą sama Pani zaaplikuje w toalecie, oraz wspomagająco elektrodami powierzchniowymi na mięśniach brzucha oraz mięśniu pośladkowym, które badacz przyklei Pani do skóry. W czasie badania będzie Pani w pełni ubrana. W pozycji leżącej na kozetce wykona Pani napięcia i rozluźnienia mięśni dna miednicy. Po zakończeniu sekwencji testowej otrzyma Pani raport wraz z konsultacją instruktora i rozpocznie Pani ćwiczenia napinania i rozluźniania mięśni dna miednicy, obserwując zmiany ich napięcia na monitorze.

d. Następnie wykona Pani napięcia i rozluźnienia mięśni dna miednicy w warunkach dynamicznych, to jest w czasie marszu, biegu w miejscu, przysiadu oraz ćwiczeń na mięśnie brzucha.

Umiejętność rozluźnienia mięśni dna miednicy będzie dla Pani przydatna w czasie korzystania z toalety (w czasie mikcji i defekacji), a umiejętność ich zaciskania pomoże Pani uniknąć nietrzymania moczu w czasie kaszlu, kichania, śmiechu, podnoszenia ciężarów, podskoków i innych czynności.

Zajęcia ruchowe: Program aktywności fizycznej obejmuje rożne formy treningu zdrowotnego m.in. treningi New Walking, ćwiczenia wzmacniające prowadzone na siłowni i/lub sali gimnastycznej. Zajęcia będą przeprowadzone 2-3 razy w tygodniu. Miejsce zajęć: AWFiS, lasy oliwskie.

Ryzyko związane z badaniami i udziałem w zajęciach ruchowych

Przez cały okres wykonywania przewidzianych protokołem badań, pozostanie Pani/Pan pod opieką personelu Laboratorium Wysiłku Fizycznego AWFiS w Gdańsku. Pobranie krwi oraz podstawowe jej oznaczenia wykona wykwalifikowany personel Laboratorium Medycznego Synevo w Gdańsku. Możliwe jest uczucie dyskomfortu podczas procedury pobrania krwi, natomiast po pobraniu mogą wystąpić zasinienia lub krwiaki.

Pozostałe badania są badaniami nieinwazyjnymi. Badania sprawności fizycznej i wydolności tlenowej, podobnie jak udział w zajęciach ruchowych może zwiększyć możliwość wystąpienia kontuzji i urazów oraz problemy zdrowotne związane z pracą narządów i układów w tym m.in. zaburzenia układu krążenia. W przypadku wystąpienia u Pani/Pana jakichkolwiek pytań i wątpliwości związanych z przeprowadzanym badaniem, nasz personel lub trener postara się na nie rzetelnie odpowiedzieć. Gdyby Pani/Pan nadal miał(a) wątpliwości odnośnie badania lub w przypadku wystąpienia zdarzeń niepożądanych procedura lub/i badanie zostanie przerwane.

Korzyści związane z badaniami i udziałem w zajęciach ruchowych

Ocena poziomu sprawności i wydolności fizycznej oraz wybranych wskaźników ryzyka wystąpienia niesprawności stanowi cenną wskazówkę obrazującą zmiany jakie zostały wypracowane w kontekście systematycznie podejmowanej aktywności ruchowej stając się normatywną oceną funkcjonowania całego organizmu.

Głównym korzystnym oddziaływaniem podejmowanej aktywności ruchowej jest wydłużenie okresu niezależności i sprawności fizycznej, a zatem i poprawa jakości życia w tym relacji społecznych. Korzyściom fizycznym i funkcjonalnym wynikających z aktywności ruchowej towarzyszą także pozytywne skutki psychologiczne w postaci lepszego nastroju, zwiększonego poczucia własnej wartości i funkcjonowania poznawczego. Aktywność ruchowa jest także wykorzystywana w prewencji pierwotnej i wtórnej, takich schorzeń jak: nadwaga, nadciśnienie tętnicze, osteoporoza, choroba zwyrodnieniowa stawów kręgosłupa, cukrzyca, stany zmniejszonej odporności na stres psychiczny. Ponadto jak pokazują metaanalizy badań epidemiologicznych opublikowane w Medicine and Science in Sports and Exercise, regularny wysiłek fizyczny obniża o około 30% ryzyko umieralności ogólnej.

Uczestnicy projektu: Kobiety i mężczyźni po 60 roku życia nie posiadający przeciwwskazań do wysiłku fizycznego.

Poufność wyników badania

Pani/Pana prywatność jest chroniona poprzez obowiązek zachowania poufności przez wszystkich, którzy są zaangażowani przy pracy z danymi i materiałem biologicznym. Osoby te są zobowiązane do przestrzegania przepisów rozporządzenia o ochronie danych osobowych z dnia 27 kwietnia 2016 r. RODO oraz Polityki Ochrony Danych Osobowych w Akademii Wychowania Fizycznego i Sportu w Gdańsku (AWFiS), określającej zasady ochrony danych osobowych w badaniach naukowych

prowadzonych w AWFiS. Po wyrażeniu zgody na badanie Pani/Pana dane, w tym dane o zdrowiu, oraz pobrany materiał biologiczny będą oznakowane specjalnym unikalnym kodem projektowym (zapisany w postaci 7 cyfrowego kodu), nadanym Pani/Panu w badaniu.
Dla potrzeb niniejszego badania utworzona zostanie elektroniczna naukowa baza danych zawierająca informacje o uczestnikach badania, stanie ich zdrowia i wynikach analiz pobranego materiału biologicznego. Naukowa baza danych nie zawiera żadnych informacji identyfikujących uczestników badania, a jedynie przypisany każdemu z nich kod projektowy. Baza danych ostatecznie posłuży do wykonania kompleksowych analiz na danych zebranych od wszystkich uczestników badania. Pani/Pana dane identyfikacyjne zostaną użyte w celu uzupełniania i aktualizowania informacji z Pani/Pana dokumentacji medycznej, do przyszłej komunikacji z Panią/Panem odnośnie obecnego badania oraz zaproszenia do przyszłych badań naukowych.

Opłaty

Uczestnictwo w badaniu jest dobrowolne i nie jest związane z gratyfikacją finansową dla Pani/Pana oraz rodziny. Nie ponosi Pani/Pan również kosztów prowadzonych badań oraz opłat związanych z udziałem w programie aktywności fizycznej i konsultacjach.

Warunki uczestnictwa

- Wiek powyżej 60 roku życia.
- Zaświadczenie od lekarza o braku przeciwwskazań do wysiłku fizycznego o charakterze treningu zdrowotnego i próby wysiłkowej.
- Ubezpieczenie od następstw nieszczęśliwych wypadków (NNW) w okresie trwania badań i zajęć ruchowych.
- Udział w badaniach oraz systematyczne uczestnictwo w prowadzonych zajęciach (dozwolona jest 3 tygodniowa absencja w prowadzonych zajęciach z przyczyn losowych).
- Zgoda na udział w projekcie.

Informacje dodatkowe

1. Po badaniach możliwa jest konsultacja wyników w kierunku profilaktyki wybranych czynników wystąpienia niesprawności z udziałem aktywności fizycznej.
2. Zachęcamy Panią/Pana do zadawania pytań, które dotyczą badania i zajęć ruchowych. Jeśli Pani/Pan będzie miał(a) dodatkowe pytania prosimy dzwonić na numer 58 5547184 (najlepiej we wtorki w godz. 15.00-16.30) lub pisać na e-mail: awfis.rek@wp.pl

ZGODA NA UDZIAŁ W BADANIU I PROGRAMIE ZAJĘĆ RUCHOWYCH

Kod uczestnika …………………………………

Dobrowolnie wyrażam zgodę na udział w badaniu i programie zajęć ruchowych:

„Aktywność fizyczna a wybrane wskaźniki ryzyka wystąpienia niesprawności u osób starszych”

Potwierdzam, że przeczytałam(em) i zrozumiałam(em) powyższą informację o badaniu i zajęciach ruchowych. Uzyskałam(em) niezbędne informacje na temat proponowanego mi badania. Miałam(em) możliwość zadawania pytań i otrzymałam(em) zadowalające odpowiedzi na te pytania. Rozumiem, że mój udział w badaniu jest dobrowolny i że mogę wycofać zgodę w dowolnym momencie.

Wyrażam zgodę na badania wymienione w powyższej informacji o badaniu.	**TAK*** ☐	**NIE** ☐
Wyrażam zgodę na udział w zajęciach ruchowych i informuję, że nie są mi znane przeciwwskazania do wymienionych badań i wysiłku fizycznego.	**TAK** ☐	**NIE** ☐
Wyrażam zgodę na dostęp do dokumentacji o moim stanie zdrowia oraz do przechowywania i wykorzystywania tych i innych informacji o mnie, dla celów naukowych, wyłącznie w formie anonimowej.	**TAK** ☐	**NIE** ☐
Wyrażam zgodę na umieszczanie moich zanonimizowanych informacji w bazach danych, prezentacjach i publikacjach naukowych.	**TAK** ☐	**NIE** ☐
Wyrażam zgodę na bezterminowe przechowywanie, w formie zakodowanej, mojego materiału biologicznego (w tym genetycznego), oraz powiązanych danych, i ich wykorzystanie w przyszłych badaniach naukowych mających na celu promowanie zdrowia w społeczeństwie.	**TAK** ☐	**NIE** ☐

Wyrażam zgodę na ponowny kontakt w przyszłości w celu uzyskania zgody na:

1. uzupełnienia i uaktualnienia moich informacji zdrowotnych **TAK** ☐ **NIE** ☐
2. zaproszenia do kolejnych badań **TAK** ☐ **NIE** ☐

**** proszę zaznaczyć ‘x’ wybraną odpowiedź***

____________________________	____________	__________________________
Imię i nazwisko uczestnika	data	podpis uczestnika

__________________________	____________	__________________________
Imię i nazwisko kierownika badania/ projektu	data	podpis kierownika badania/ projektu

Dokument sporządzono w dwóch jednobrzmiących egzemplarzach, po jednym dla uczestnika i organizatora projektu.
