# Supplementary material for: Effect of short-term exercise with different programs on prevention of sarcopenia in postmenopausal women: A Quasi-Randomized Controlled Trial
Source: PLoS One. 2025 Sep 30;20(9):e0333171. doi: 10.1371/journal.pone.0333171 (PMC12483237; doi:10.1371/journal.pone.0333171)
Supplement: S7 File — (PDF) [file pone.0333171.s007.pdf]

Information about the study and exercise programme and informed consent of the participant

Study assumptions:

Study/project topic: Physical activity and selected risk indicators for disability in older people.

Organizer: Academy of Physical Education and Sport in Gdansk. Study/project manager-Dr. Hab. Zbigniew Ossowski, prof. AWFiS,

Miejsce realizacji radi: Akademia Wychowania Fizycznego i Sportu (AWFiS), ul. Kazimierza Gorskiego 1, Exercise Laboratory.

Research procedure:

Studies include:

- assessment of physical fitness and selected health indicators i.e. Assessment of motor capacity, aerobic capacity and risk factors for falls, anthropometric and blood pressure studies, determination of pain threshold, study of health behaviours (including analysis of diet) and cognitive function.
- Blood laboratory tests: baseline (morphology, lipid profile, glucose, keratin kinase, CRP protein, ALT, aspate, creatinine, albumin, uric acid, total protein, calcium, electrolytes).
- Gut microflora testing.

Your coded samples of biological material (blood and faeces) will be stored at very low temperatures in a room to which only authorised persons have access to carry out their additional biochemical, proteomic and genetic labelling. Your biological material will be stored indefinitely until it is completely used up. If the material is found to be unsuitable for scientific research or if you withdraw your consent for its use, the material will be destroyed in accordance with the procedures in force at AWFIS in Gdansk.

Interested women may also participate in a pelvic floor muscle activity study using surface electromyography in the direction of urinary incontinence. Your participation in the study will include:

a. Filling in the so-called Subject metrics, including information about your age, weight, family problems with urinary disorders, etc.
b. *Completing the Incontinence Impact Questionnaire (IIQ); You will determine if, in what situations and to what extent you experience incontinence.*
c. Assessment of pelvic floor muscle function using surface electromyography with biofeedback training. Pelvic floor muscle function will be assessed using a vaginal electrode, which you will apply yourself in the toilet and, in addition, surface electrodes on your abdominal muscles and gluteal muscles, which the researcher will attach to your skin. You'll be fully clothed during the exam. In the supine position, you will perform tension and relaxation of the pelvic floor muscles. At the end of the test sequence, you will receive a report together with a consultation with the instructor and you will begin exercises to tighten and relax the pelvic floor muscles, observing the changes in their tension on the monitor.

d. You will then perform tension and relaxation of the pelvic floor muscles under dynamic conditions, i.e. during walking, standing running, squats and abdominal exercises.

The ability to relax your pelvic floor muscles will help you when using the toilet (urinating and defecating), and the ability to tighten them will help you avoid incontinence when coughing, sneezing, laughing, lifting weights, jumping and other activities.

Zajecia ruchowe: The physical activity program includes various forms of health training. And. New Walking, strengthening exercises in the gym and/or gym. Classes will be held 2-3 times a week. Place of activity: AWFiS, Lasy olivskie.

Risks associated with research and participation in physical activity

Throughout the protocol, you will remain under the care of the staff of the AWFIS Physical Exercise Laboratory in Gdansk. Blood collection and basic analysis will be performed by qualified personnel at Synevo Medical Laboratory in Gdansk. You may feel discomfort during the blood draw procedure, and bruising or haematoma may occur after the blood draw.

The remaining studies are non-invasive. Physical fitness and aerobic capacity tests, as well as participation in physical activities, may increase the possibility of injuries and health problems related to the functioning of organs and systems, including those of the body. And. Vascular disorders. If you have any questions and concerns about the survey, our staff or trainer will try to answer them accurately. If you still have doubts about the study or if adverse events occur, the procedure and/or the study will be discontinued.

Benefits of research and physical activity

The assessment of the level of fitness and physical fitness and the selected indicators of the risk of impairment is a valuable indication of the changes that have been developed in the context of systematic exercise, becoming a normative assessment of the functioning of the whole organism. The main beneficial effect of physical activity is a prolonged period of independence and physical fitness, and therefore an improvement in the quality of life, including social relations. The physical and functional benefits of physical activity are also accompanied by positive psychological effects in the form of improved mood, self-esteem and cognitive functioning. Physical activity is also used in the primary and secondary prevention of conditions such as: overweight, high blood pressure, osteoporosis, degenerative diseases of the joints of the spine, diabetes, states of reduced immunity to mental stress. In addition, meta-analyses of epidemiological studies published in Medicine and Science in Sports and Exercise show that regular exercise reduces the risk of overall mortality by about 30%.

Project participants: Women and men over 60 years of age with no contraindications to physical exertion.

Confidentiality of study results

Your privacy is protected by the obligation of confidentiality of all those involved in working with biological data and material. These persons are obliged to comply with the provisions of the Regulation on the protection of personal data of 27 April 2016. RODO and the Personal Data Protection Policy of the Academy of Physical Education and Sport in Gdansk (AWFiS), specifying the principles of personal data protection in scientific research

Conducted in AWFIS. Once you have consented to the study, the data, including health data, and the biological material collected will be marked with a special unique design code (written as a 7-digit code) given to you in the study.
For the purposes of this study, an electronic scientific database will be set up containing information on the study participants, their state of health and the results of analyses of the collected biological material. The scientific database does not contain any information identifying the study participants, only a project code assigned to each participant. The database will eventually be used for comprehensive analyses of data collected from all study participants. Your identification data will be used to complete and update information in your medical records, for future communication with you about the current study and to invite you to future scientific research.

Fees

Participation in the study is voluntary and does not involve financial compensation for you or your family. You also do not bear the costs of the research carried out and the fees associated with participation in the physical activity programme and consultations.

Conditions of participation

- Over 60 years of age.
- Certificate from a doctor stating that there are no contraindications to physical exertion of the nature of health training and exercise tests.
- Insurance against the consequences of unfortunate accidents (NNW) during the course of studies and training.
- Participation in studies and systematic participation in classes (3 weeks absence from classes for random reasons is allowed).
- consent to participate in the project.

Additional information

1. After the studies, it is possible to consult the results towards the prevention of selected factors of disability with the participation of physical activity.
2. We encourage you to ask questions that relate to the study and exercise. If you have any further questions, please call 58 554 7184 (preferably on Tuesdays at 10:00 a.m.). 15.0016. 30) or by e-mail: awfis.rek @ wp. EN

Consent to participate in the study and exercise programme

Participant code ……………………………………………….. ..

I voluntarily agree to participate in the study and exercise program:

Physical activity and selected risk indicators for disability in the elderly

I confirm that I have read and understood the above information about the study and exercise activities. I have received the necessary information about the proposed study. I had the opportunity to ask questions and received satisfactory answers to those questions. I understand that my participation in the study is voluntary and that I may withdraw my consent at any time.

I agree to the studies mentioned in the above study information. **Yes** * ☐ **No.** ☐

I agree to participate in exercise and inform you that I am not aware of any contraindications to the mentioned studies and physical exertion. **Yes** ☐ **No.** ☐

I consent to access my health records and to the storage and use of this and other information about me for the following purposes:
Scientific, anonymously only. **Yes** ☐ **No.** ☐

I consent to the inclusion of my anonymized information in scientific databases, presentations and publications. **Yes** ☐ **No.** ☐

I consent to the indefinite storage, in coded form, of my biological material (including genetic), and related data, and their
Use in future scientific research to promote
Health in society. **Yes** ☐ **No.** ☐

I agree to be contacted again in the future for consent to:

1. Completing and updating my health information **Yes** ☐ **No.** ☐
2. Invitations to follow-up studies **Yes** ☐ **No.** ☐

***** Please mark 'x' to the selected answer***

____________________________	___________	__________________________
Name of participant	Date	Participant signature

___________________________	___________	__________________________
Name of manager<br>Research/project	Date	Manager's signature<br>Research/project

The document is drawn up in duplicate, one for the participant and one for the project organiser.
