# Supplementary material for: Effect of short-term exercise with different programs on prevention of sarcopenia in postmenopausal women: A Quasi-Randomized Controlled Trial
Source: PLoS One. 2025 Sep 30;20(9):e0333171. doi: 10.1371/journal.pone.0333171 (PMC12483237; doi:10.1371/journal.pone.0333171)
Supplement: S8 File — (DOCX) [file pone.0333171.s008.docx]

**INFORMATION ABOUT THE STUDY**

**Topic:** Physical activity and selected risk indicators of disability in the elderly.

**Purpose of research:**

1. Determination of the influence of directed physical activity on cognitive functions and prevention of the risk of sarcopenia, overweight and falls in the examined persons.

2. Determination of the influence of various training programs on the activity of the pelvic floor muscles.

3. Determining the relationship between physical fitness and selected blood biomarkers in the elderly.

Blood serum was labeled and frozen.

**Place of research:**

Academy of Physical Education and Sport (AWFiS), ul. Kazimierza Górskiego 1, Laboratory of Physical Effort.

**Methods:**

1. Registration of the level of physical fitness and selected risk factors of disability in the elderly towards sarcopenia, falls, stress urinary incontinence, obesity and cardiovascular diseases.

a. arm and leg strength: hand dynamometer (Hand Grip, Biodex)

b. static and dynamic balance (Zebris platform)

c. cognitive tests: (WST and Trail Making Test A, B)

d. measurement of body composition and selected anthropometric indicators (InBody 720 composition analyzer, tailor's tape measure, goniometer, anthropometer)

e. functional fitness (Senior Fitness Test, Gait speed, "Get up and go" test)

f. aerobic capacity (Oksykon)

g. gait (Footscan)

h. reaction speed and motor coordination (Blink)

2. Measurement of blood pressure.

3. Study of pelvic floor muscle activity using surface electromyography for urinary incontinence.

4. Laboratory analysis of blood (basic determinations): morphology, lipid profile, glucose, keratin kinase, CRP protein, ALT, AST, creatinine, albumin, uric acid, total protein, calcium, electrolytes).

6. Determination of selected blood biomarkers (Luminex).

7. Analysis of diet and eating habits, diet logs (Nuvero program).

8. Assessment of health behavior, quality of life, cognitive functions and the level of physical activity (International Physical Activity Questionnaire - short version, psychological tests-COG).

**Inclusion criteria:**

• Women over 60 years of age, with no contraindications to exercise.

• Signing consent to participate in research and physical activity program.

**Organization of research and information about physical activities:**

Participants from the two EG groups began to implement health training: Nordic Walking - HIIT (EG1) and resistance training (EG2). Three times a week, 60 minutes each time, for a period of 12 weeks. The CG group did not take the courses of awfis.
